# Impact of minimum distance constraints on sheet metal waste for plasma cutting

**DOI:** 10.1371/journal.pone.0292032

**Published:** 2023-09-27

**Authors:** Matheus Francescatto, Alvaro Luiz Neuenfeldt Júnior, Elsa Silva, João Carlos Furtado, Dani Bromberger

**Affiliations:** 1 Innovation and competitiveness group, Production Engineering post-graduation program, Federal University of Santa Maria, Santa Maria, Rio Grande do Sul, Brazil; 2 Systems Engineering and Operational Research (SEOR), Centro ALGORITMI, University of Minho, Guimarães, Portugal; 3 Production Engineering post-graduation program, Federal University of Santa Maria, University of Santa Cruz do Sul, Rio Grande do Sul, Brazil; Cyprus International University Faculty of Engineering: Uluslararasi Kibris Universitesi Muhendislik Fakultesi, TURKEY

## Abstract

We approached the two-dimensional rectangular strip packing problem (2D-SPP), where the main goal is to pack a given number of rectangles without any overlap to minimize the height of the strip. Real-life constraints must be considered when developing 2D-SPP algorithms to deliver solutions that will improve the cutting processes. In the 2D-SPP literature, a gap related to studies approaching constraints in real-life scenarios was identified. Therefore, the impact of real-life constraints found in the plasma cutting process in sheet metal waste was analyzed. A mathematical model from the literature was modified to obtain packing arrangements with plasma cutting constraints. The combination of size and number of rectangles, as well as strip width, was the main factor that affected the packing arrangement, limiting the allocation of rectangles and generating empty spaces. In summary, considering the sheet metal waste context, instances with smaller widths should be avoided in practical operations for high minimum distance constraint values, returning the worst packing arrangements. For low minimum distance constraint values, smaller width instances can be used in practical operations, as the packing arrangement is acceptable. Finally, this article can reduce material waste and enhance the cutting process in the sheet metal industry, by showing packing characteristics which lead to higher amounts of raw material waste.

## Introduction

In the two-dimensional rectangular strip packing problem (2D-SPP), a determined number of rectangles must be packed in a rectangular strip without overlap. Furthermore, the strip width is known, and the strip height is considered infinite, being characterized as an open dimension problem [1]. Therefore, the main 2D-SPP goal is to minimize the strip height. Since the 1980s, the 2D-SPP has been widely studied due to its practical applications in reducing waste, lowering operational costs in industrial production processes, and mitigating the environmental impact of raw material transformation activities. However, to achieve the 2D-SPP objectives, practical constraints must be considered, impacting the algorithms directly. From constraints found in different industries, the algorithms are created for a specific scenario, resulting in operational gains. For a more detailed understanding of the 2D-SPP characteristics and definitions, refer to [2].

To design solutions to be applied in realistic scenarios, constraints found in 2D-SPP practical applications must be considered. In this research, two specific sheet metal plasma cutting constraints found in the steel industry were considered, the minimum distance between the rectangles and the minimum distance between the rectangles and the strip edges. The minimum distance between rectangles is necessary due to the plasma-generated heat, where close rectangles are damaged, suffering dimensional deformation. Furthermore, the minimum distance between the rectangles and the edges relates to damage caused by sheet metal fabrication, transportation, and storage, which can prevent areas near the edges from being used. Both minimum distance constraints are named “plasma cutting constraints” (PCC).

The sheet metal plasma cutting process has numerous advantages, enabling high cutting speeds and increased productivity due to its efficiency [3]. Also, sheet metal materials, including steel, stainless steel, and aluminum can be cut, due to the sheet manufacturing processes accurate control to achieve desired proprieties [4–6]. During the cutting, the plasma generates a narrow heat-affected zone, preserving the structural integrity of the sheet metal and producing cuts with minimal dross and slag, reducing the need for additional machining operations [7]. Also, the cut precision is achieved through computer numerical control, reducing material waste by minimizing errors and optimizing material usage. However, even considering the plasma cutting precision, depending on the packing layout, empty spaces can be found between rectangles, originating raw material waste.

In addition, when considering the 2D-SPP found in the sheet metal plasma cutting, the strip’s height is minimized, being crucial for optimizing material utilization and reducing raw material waste [8, 9]. Consequently, the cutting process is more efficient with reduced costs. Also, overlapping rectangles during the cutting process are not allowed, avoiding discarding rectangles with incorrect geometric dimensions [2, 10].

The objective of this research is to verify the impact of PCC in sheet metal waste, according to the instances’ characteristics, improving the economic efficiency of plasma cutting operations. Different approaches can be used to solve the PCC. In this research, our contribution is to solve the PCC by modifying the mixed-integer linear programming (MILP) formulation proposed by [9]. Also, the solutions obtained with the model are packing arrangements already with the PCC, which facilitates the representation of the solution. Therefore, the sheet metal plasma can be used immediately without pre- or post-processing, avoiding possible operator errors and increasing the processing speed. In addition, the modification proposed in the MILP is only used to find packing layouts with the PCC, enabling an in-depth analysis of the raw material waste, not being considered a main contribution in the present research.

Recently, practical applications have been addressed by other cutting and packing problems, such as the cutting stock problem in concrete [11], construction [12], mattress [13], glass manufacturing [14], and paper production [15] industries. In addition, multi-objective lot sizing and cutting stock approaches were inspired by practical applications [16]. Furthermore, the bin packing problem was analyzed based on real-life scenarios, considering time windows [17, 18], item fragmentation [19], and applied in a spare parts company [11]. Finally, for the knapsack problem, realistic container loading constraints were considered in [20]. Furthermore, a bibliometric review of the 2D-SPP and a detailed discussion of the constraints found in the 2D-SPP literature were presented in [21]. From the articles above, compared to other cutting and packing problems, the 2D-SPP literature needs more practical studies, as shown in Neuenfeldt et al. [21]. In addition, from the 2D-SPP literature, only seven articles approached constraints based on real-life scenarios [22–28].

Researchers in [22–25] have approached the 2D-SPP from a multi-objective perspective, focusing on minimizing both raw material waste and the number of cuts in the production process. In [22], the authors proposed a coding scheme that combines a complete representation of layout patterns with a hyperheuristics approach. For [23], some of the best-known multi-objective evolutionary algorithms were applied, implementing hyperheuristic-based encodings. In [24], a dedicated multi-objective evolutionary algorithm was developed. Additionally, [25] employed a Greedy Randomized Adaptive Search Procedure algorithm to generate the initial population for the multi-objective algorithm. To enhance the performance of the multi-objective algorithm, four distinct positioning heuristics were integrated: next-fit, a modified version of next-fit, best-fit, and first-fit.

A new algorithm based on a column generation technique, considering the 3-stage guillotine cutting constraint was proposed in [26]. A coiled stock material used in practical cutting operations was considered. In addition to the 3-stage guillotine cutting constraint, the number of available cutting blades was restricted. For [27], the authors focused on addressing the scoring restriction in cardboard cutting machines, where rectangles needed to be packed in the strip while adhering to a minimum scoring distance specified by the scoring machines. The authors developed an exact polynomial-time algorithm integrated with a packing heuristic, comparing the algorithm with two greedy heuristics.

In the production of corrugated cardboard, an additional constraint known as the sequencing constraint must be considered. This constraint arises from the restrictions imposed by dampers, which allow only two rectangles to be processed simultaneously. To address this constraint, [28] proposed the development of two distinct algorithms that leverage the graphical characterization of viable solutions. The first algorithm adopts a two-phase procedure based on a mixed-integer linear programming model, while the second algorithm follows a combinatorial approach to solve the sequencing constraint.

Also, for the 2D-SPP, new exact methods and algorithms were proposed in recent years, without considering practical constraints. In [29], a methodology called Positions and Covering was developed, providing exact and previously unknown solutions for literature instances with validated results and effectiveness for small and medium-size instances. Additionally, [30] proposed a novel algorithm based on deep reinforcement learning, achieving superior optimization results, and demonstrating high solution efficiency, generalization, and potential for practical applications.

Considering practical 2D-SPP applications, articles approached raw material waste and the number of cuts [22–25], coiled stock material [26], cardboard cutting machines [27], and corrugated cardboard [28]. Therefore, a minimum distance constraint was not necessary for the practical applications addressed. The PCC directly impacts the packing of rectangles, creating a different layout when compared with the 2D-SPP practical articles. Furthermore, the minimum score distance [27] is the closest approach to the PCC. However, the minimum score constraint only applies when rectangles are packed horizontally, while the PCC are applied to rectangles packed both vertically and horizontally.

From the practical 2D-SPP research context, prior articles have not addressed sheet metal plasma cutting. Therefore, a gap was found in the 2D-SPP literature related to approaching real-life constraints found in plasma sheet metal cutting. As a result, this article contributes to both the academic and practical 2D-SPP area by addressing sheet metal plasma cutting with real-life constraints, being, to the best of the author’s knowledge, the first approach able to verify the PCC impact on raw material waste.

Furthermore, industries involved in sheet metal plasma cutting, where the PCC are commonly found, can use this articles’ findings to enhance the cutting process. By understanding packing characteristics that contribute to increased raw material waste, industries can improve production planning minimizing waste in the cutting process. Consequently, this optimization leads to economic advantages and minimizes environmental issues, due to the reduction of raw material utilized.

The remainder of this paper is structured as follows. Section 2 presents the problem statement with the PCC formulation. Section 3 shows the scenarios considered to categorize the instances analyzed, defines an indicator used to evaluate solutions, and describes the impact on sheet metal waste considering the PCC equal to 1, 2, and 3, as well as a summary containing the main findings. Finally, a conclusion about the study is presented in Section 4. Additionally, S1 Appendix introduces the basic notation used for reference and S2 Appendix provides tables containing information on the instances used and the results found.

## Problem statement

During the plasma cutting operation, the heat from the electric arcs of ionized gas is released by a plasma column. Subsequently, the plasma melts the metal part, causing the cut. One advantage is to improve work control, eliminating the need for adjusting flammable gas mixtures. It is safer to perform and the risk of deformation in the cutting area caused by thermal propagation is reduced. However, even if the deformation risk due to thermal propagation is lower, a minimum distance between the rectangles must be considered based on the heat-affected zone generated during the cut. Adhering to this minimum distance ensures the rectangles adjacent to the one being cut remain unaffected by thermal propagation, eliminating subsequent machining operations and reducing overall process costs. Consequently, a minimum distance between rectangles, defined as *β*, is the first PCC input parameter.

In addition, *β* has a direct relation with the kerf width found in sheet metal plasma cutting. The kerf width represents cut width or groove produced, being influenced by factors including cutting speed, power settings, material thickness, standoff distance, plasma gas composition, nozzle design, and material properties [3]. Therefore, for different plasma cutting parameters, the kerf width, and consequently *β*, vary. Table 1 shows approximate *β* values for different plasma cutting configurations.

**Table 1:** Approximate *β* values for different plasma cutting parameters [31].

Steel type	Thickness (mm)	Electric current (A)	Approximately *β* value (cm)
Carbon steel	1.0	45	1.0
	3.0	65	1.6
	8.0	65	1.9
	20.0	85	3.0
Stainless steel	1.0	45	1.1
	3.0	65	1.5
	8.0	65	1.9
	20.0	85	2.5
Aluminum	1.0	45	1.5
	3.0	65	1.9
	8.0	65	2.0
	20.0	85	2.6

Furthermore, metal sheets can be susceptible to damage and deformation during fabrication, transport, and storage, particularly at the sheet edges, making the area unusable. Therefore, a minimum distance between the rectangles and the edges of the sheet, defined as *δ*, is the second PCC input parameter. Also, factors impacting δ are particular to the industries where plasma sheet metal cutting is found, varying according to production process characteristics, the equipment used, operator qualification, and raw material suppliers’ quality. Both *β* and *δ* are the PCC, being include in the adapted model.

### Adapted MILP model for the strip packing problem with PCC

**Fig 1:**
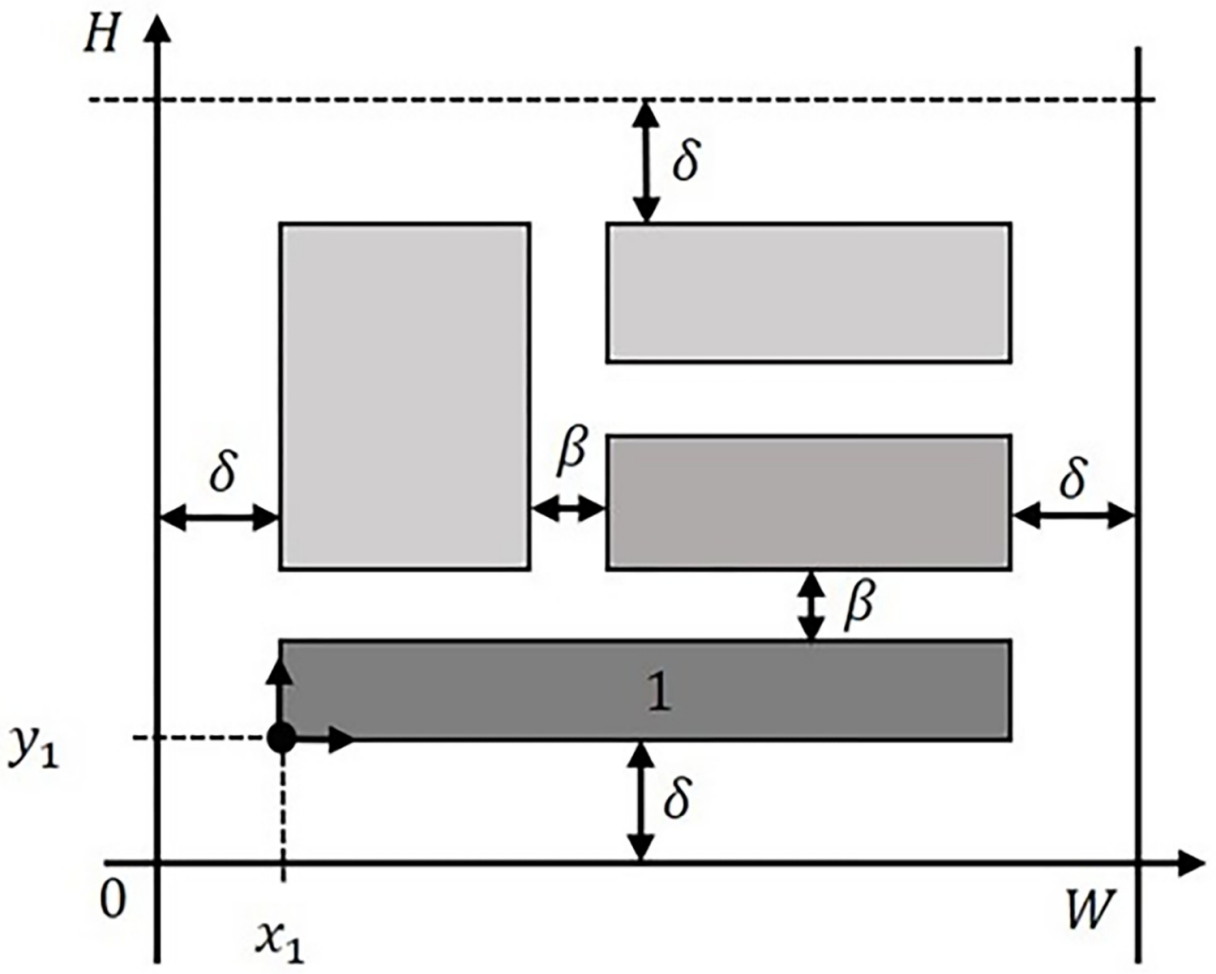
Example of packing arrangement considering the PCC.

An adaptation of the mixed-integer linear programming formulation proposed by [9], considering rectangle rotation and the PCC, was used. A set of rectangles *I* = {1,2,…,*n*} are packed without overlap to minimize the total strip height (*H*). The strip width (*W*) and the rectangles’ characteristics are previously known, representing an offline packing. Each rectangle is defined by two dimensions, height (*h*_*i*_) and width (*w*_*i*_), where *i*∈*I*. The position of rectangles inside the strip is determined by the coordinates *x*_*i*_ and *y*_*i*_. A bottom-left approach is considered during the packing process, where the rectangle’s bottom-left corner is positioned in the defined *x*_*i*_ and *y*_*i*_ coordinates, as shown in Fig 1 for rectangle 1.

An alternative approach to solve the PCC involves incorporating adjustments into the selected instance. This includes adding *β*/2 to both *h*_*i*_ and *w*_*i*_, as well as introducing a constraint in the model which requires *x*_*i*_ and *y*_*i*_ to be greater than or equal to *δ*/2. Additionally, *δ*/2 needs to be subtracted from the values of *H* and *W*. However, this approach requires data pre-processing to update the instance characteristics, and data post-processing to adapt the rectangles’ dimensions to the original format, complicating the solution representation.

The adapted mixed-integer linear programming from [9], considering 90° rectangle rotation, the PCC, and parameters *β* and *δ* is described next. For the rotation of rectangles, a binary decision variable *α*_*i*_ was defined, as being equal to one if the rectangle is not rotated (*α*_*i*_ = 1), maintaining the original rectangle dimensions. If the rectangle is rotated, then *α*_*i*_ = 0, and its dimensions are changed by swapping *h*_*i*_ with *w*_*i*_. Also, *z*_1*ij*_, *z*_2*ij*_, *z*_3*ij*_, and *z*_4*ij*_ are binary decision variables used with the Big-M method to avoid rectangle overlapping, where *z*_1*ij*_ = 1 determines if the rectangle is allocated at the left of a rectangle already positioned, *z*_2*ij*_ = 1 determines if the rectangle is allocated at the right of a rectangle already positioned, *z*_3*ij*_ = 1 determines if the rectangle is allocated below a rectangle already positioned, and *z*_4*ij*_ = 1 determines if the rectangle is allocated above a rectangle already positioned.


minimizeH (1)

$$subject\;to\;x_{i}+w_{i}+h_{i}{(1-\alpha }_{i})\leq W-\delta \;\forall i\in I,$$ (2)

$$y_{i}+h_{i}\alpha_{i}+w_{i}{(1-\alpha }_{i})\leq H-\delta \;\forall i\in I,$$ (3)

$$x_{i}+h_{i}{(1-\alpha }_{i})+w_{i}\alpha_{i}+\beta \leq x_{j}+M(1-z_{1ij})\;\forall i,j\in I,i\neq j,$$ (4)

$$x_{j}+h_{j}{(1-\alpha }_{j})+w_{j}\alpha_{j}+\beta \leq x_{i}+M(1-z_{2ij})\;\forall i,j\in I,i\neq j,$$ (5)

$$y_{i}+h_{i}\alpha_{i}+w_{i}{(1-\alpha }_{i})+\beta \leq y_{j}+M(1-z_{3ij})\;\forall i,j\in I,i\neq j,$$ (6)

$$y_{j}+h_{j}\alpha_{j}+w_{j}{(1-\alpha }_{j})+\beta \leq y_{i}+M(1-z_{4ij})\;\forall i,j\in I,i\neq j,$$ (7)

$$z_{1ij}+z_{2ij}+z_{3ij}+z_{4ij}=1\;\forall i,j\in I,i\neq j,$$ (8)

$$x_{i}\geq \delta \;and\;y_{i}\geq \delta \;\forall i\in I,$$ (9)

$$\alpha_{i},z_{1ij},z_{2ij},z_{3ij},z_{4ij}\in \{0,1\}\;\forall i,j\in I.$$ (10)

The objective function (1) is related to strip height *H* minimization. Constraint (2) refers to the positioning of the rectangles within the strip, meaning the coordinate *x*_*i*_ added to *w*_*i*_ or *h*_*i*_ cannot exceed *W*−*δ*. Similarly, in constraint (3), the coordinate *y*_*i*_ added to *h*_*i*_ or *w*_*i*_ cannot exceed *H*−*δ*. Constraints (4), (5), (6), and (7) ensure that there is no overlap between rectangles by using the Big-M method. Constraint (8) guarantees that only one constraint between (4), (5), (6), and (7), is valid for any two rectangles. Thus, during the packing process, the rectangles’ coordinates, *x*_*i*_ and *y*_*i*_, added to the corresponding rectangle dimension, *w*_*i*_ or *h*_*i*_, as well as *β*, are considered to determine the final rectangle position. Constraint (9) dictates *x*_*i*_ and *y*_*i*_ must be greater or equal to *δ*, ensuring the rectangles are placed inside the strip, whilst respecting *δ*. Finally, constraint (10) characterizes the values which the Boolean variables can assume.

### Lower bound for the strip packing problem with PCC

To evaluate the solutions obtained with the modified mixed-integer linear programming formulation, a lower bound considering the PCC must be considered. A lower bound for the 2D-SPP was proposed by [8], combining two simple bounds derived from geometric considerations. The first bound (*L*_*h*_) relates to the height of the tallest rectangle, *L*_*h*_ = *max*_*j* = 1,…,*n*_{*h*_*j*_}, and the second bound (*L*_*c*_) relates to rectangles’ dimension and *W*, also called continuous lower bound, shown in Eq (11). Depending on the instance considered, both *L*_*h*_ and *L*_*c*_ can be inaccurate, where the absolute worst-case performance ratio is close to zero for larger instances (*L*_*h*_) and close to zero for wider strips (*L*_*c*_). An improved bound (*L*_0_) was proposed in [8], combining both bounds (*L*_*h*_ and *L*_*c*_), as shown in Eq (12).


$$L_{c}=\frac{\sum_{i\in I}w_{i}h_{i}}{W}$$ (11)

$$L_{0}=max(L_{h},L_{c})$$ (12)

**Fig 2:**
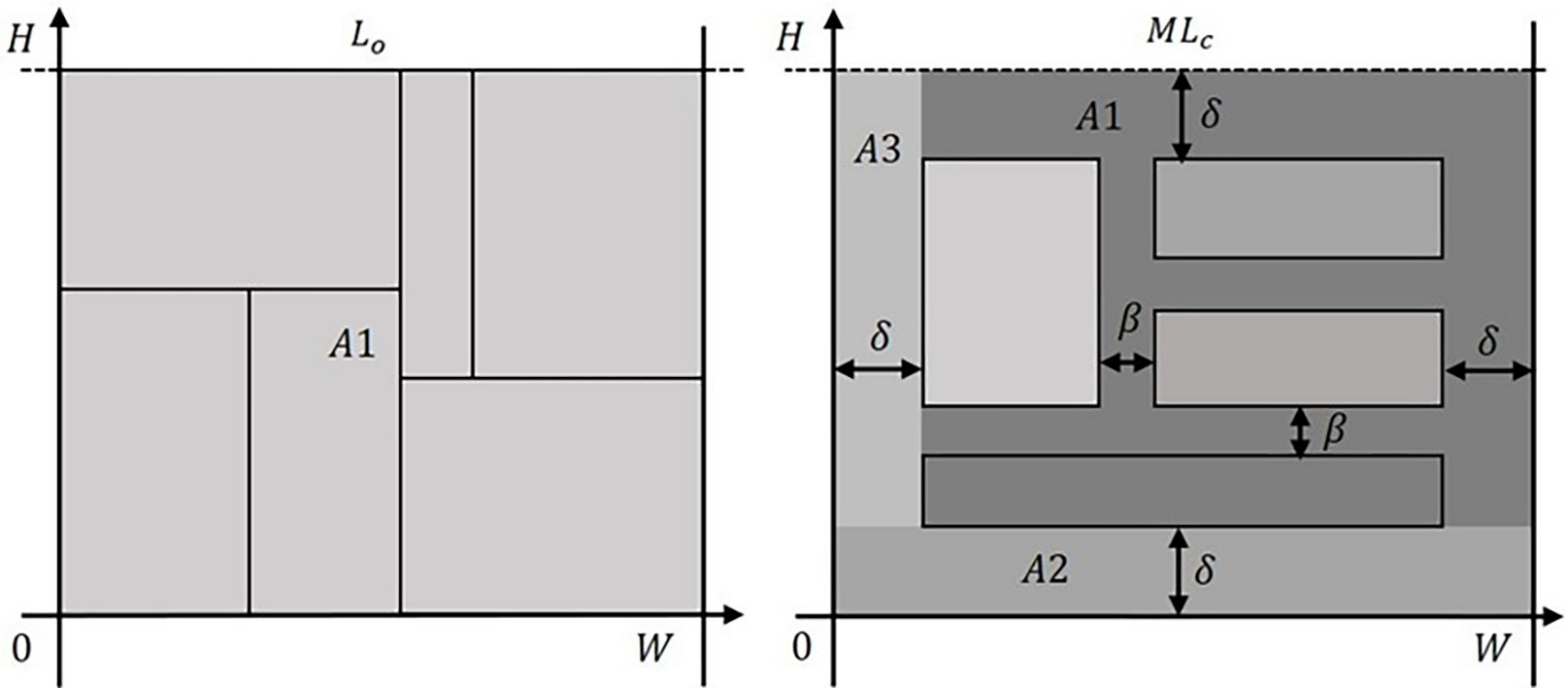
*L*_*c*_ and *ML*_*c*_ comparison.

The *L*_0_ is effective when dealing with zero waste instances (see [32]), which originates a packing arrangement without any empty space between rectangles. However, considering the PCC, the *L*_0_ becomes imprecise, since *β* and *δ* were not considered in the original *L*_0_ formulation. Also, by modifying the instance characteristics by adding *β*/2 to *h*_*i*_ and *w*_*i*_, the *L*_0_ remains imprecise, since *δ* is not considered. An adaptation of the *L*_0_ including the PCC is proposed, defined as *ML*_0_ and is composed of two bounds. The first bound (*ML*_*h*_) is related to the tallest rectangle, *ML*_*h*_ = *max*_*j* = 1,…,*n*_{2*δ*+*h*_*j*_}, and the second bound (*ML*_*c*_) is related to *h*_*i*_, *w*_*i*_, *W*, *β*, and *δ*. Fig 2 shows a comparison between *L*_*c*_ and *ML*_*c*_.

Only one area (*A*1) was considered in *L*_*c*_, based on the rectangles’ dimensions, represented by Eq (11). *ML*_*c*_ is composed of three different areas, *A*1, *A*2, and *A*3. *A*1 relates to the rectangles packed into the strip, as well as *β* and *δ*, shown in Eq (13). Also, *β* and *δ* must be equal for the *ML*_*c*_ determination.


$$A1=\sum_{i\in I}\left(h_{i}+\beta )(w_{i}+\beta \right)$$ (13)

The strip bottom position and left area are represented by *A*2 and *A*3, respectively, as shown in Eq (14) and Eq (15).


A2=Wβ (14)

$$A3=\left(\frac{A1}{W-\beta }\right)\beta$$ (15)

In Eq (16), the *ML*_*c*_ is obtained with *A*1, *A*2, and *A*3.


$$ML_{c}=\frac{(\frac{A1}{W-\beta })\beta +W\beta +\sum_{i\in I}\left(h_{i}+\beta )(w_{i}+\beta \right)}{W}$$ (16)

Finally, the *ML*_0_ is determined, combining *ML*_*h*_ and *ML*_*c*_, as shown in Eq (17).


$$ML_{0}=max(ML_{h},ML_{c})$$ (17)

A numerical example, considering all values in millimeters, illustrates the *ML*_0_. Let *W* = 10 and *β* = *δ* = 1. The number of rectangles is 7 and the rectangles’ geometric dimensions are: *w*_1_ = *w*_2_ = *w*_3_ = *w*_4_ = 2; *w*_5_ = *w*_6_ = 3; *w*_7_ = 1;, *h*_1_ = *h*_2_ = 15; *h*_3_ = *h*_4_ = 12; *h*_5_ = *h*_7_ = 9; and *h*_6_ = 8. From Eqs (13–15), *A*1 = 270, *A*2 = 10, and *A*3 = 30, respectively. Therefore, *ML*_*h*_ = 17 and *ML*_0_ = 31.

## Computational experiments

**Fig 3:**
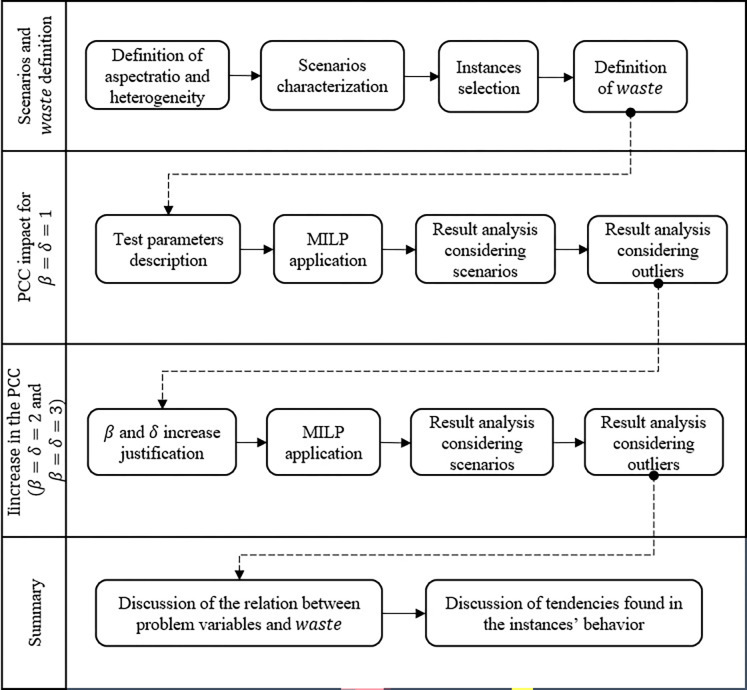
Computational experiments framework.

In this section, the objective is to analyze the PCC impact in the sheet metal waste context considering the packing arrangement found, therefore the adapted MILP model performance was not evaluated. Section 3.1 presents the scenarios used to evaluate the PCC. Section 3.2 shows the results obtained with the PCC parameters *β* and *δ* equal to 1. Section 3.3 presents the results considering an equal increase in the PCC (*β* = *δ* = 2 and *β* = *δ* = 3). Finally, in Section 3.4, a summary containing the main findings is shown. Also, Fig 3 shows a framework to summarize the computational experiments’ steps.

### Scenarios and *waste* definition

Considering the geometric complexity, scenarios were developed to analyze the impact of parameters *β* and *δ* in the solutions. Therefore, the main parameters used to evaluate the geometric complexity were the aspectratio (*ar*) and the heterogeneity (*ht*). The *ar* is used to identify and determine variations in rectangles dimensions [33], utilizing the strip geometric characteristics, higher (*D*_1_) and lower (*D*_2_) dimensions (based on *W* and *ML*_0_), and each rectangle *i* geometric characteristics: higher (*d*_1*i*_) and lower dimension (*d*_2*i*_). Additionally, the number of rectangles (*n*) from each instance is used, as shown in Eq (18). For higher values of *ar*, the difference between *d*_1*i*_ and *d*_2*i*_ of each rectangle increases, resulting in an instance of high geometric complexity [34].


$$ar=(D_{1}/D_{2})/[\sum_{i\in I}(d_{1i}/d_{2i})/n]$$ (18)

Heterogeneity (*ht*) refers to the diversification of rectangles in each instance. Furthermore, *ht* is obtained by the proportion of different rectangles, based on the absolute dimensions (*w*_*i*_ and *h*_*i*_), as shown in Eq (19), where the number of different rectangles (*nt*) is divided by *n*. Also, by the orientation constraint, rectangles with the same absolute dimensions in an instance, rotated or not, are considered equal (e.g., a rectangle with *w*_1_ = 6 and *h*_1_ = 3 is considered equal to a rectangle with *w*_2_ = 3 and *h*_2_ = 6). The maximum value for *ht* is 1, implying all rectangles are different in an instance.


ht=nt/n (19)

Both parameters, *ar* and *ht*, are related to the geometric characteristics of each instance, which are used to define the scenarios considered. Table 2 shows the scenarios characterization.

**Table 2:** Scenarios characterization.

Scenario	*ht*	*ar*
1	0≤*ht*≤0.33	0≤*ar*≤0.50
2	0≤*ht*≤0.33	0.50<*ar*≤1.00
3	0≤*ht*≤0.33	1.00<*ar*≤2.00
4	0≤*ht*≤0.33	2.00<*ar*
5	0.33<*ht*≤0.66	0≤*ar*≤0.50
6	0.33<*ht*≤0.66	0.50<*ar*≤1.00
7	0.33<*ht*≤0.66	1.00<*ar*≤2.00
8	0.33<*ht*≤0.66	2.00<*ar*
9	0.66<*ht*≤1.00	0≤*ar*≤0.50
10	0.66<*ht*≤1.00	0.50<*ar*≤1.00
11	0.66<*ht*≤1.00	1.00<*ar*≤2.00
12	0.66<*ht*≤1.00	2.00<*ar*

**Fig 4:**
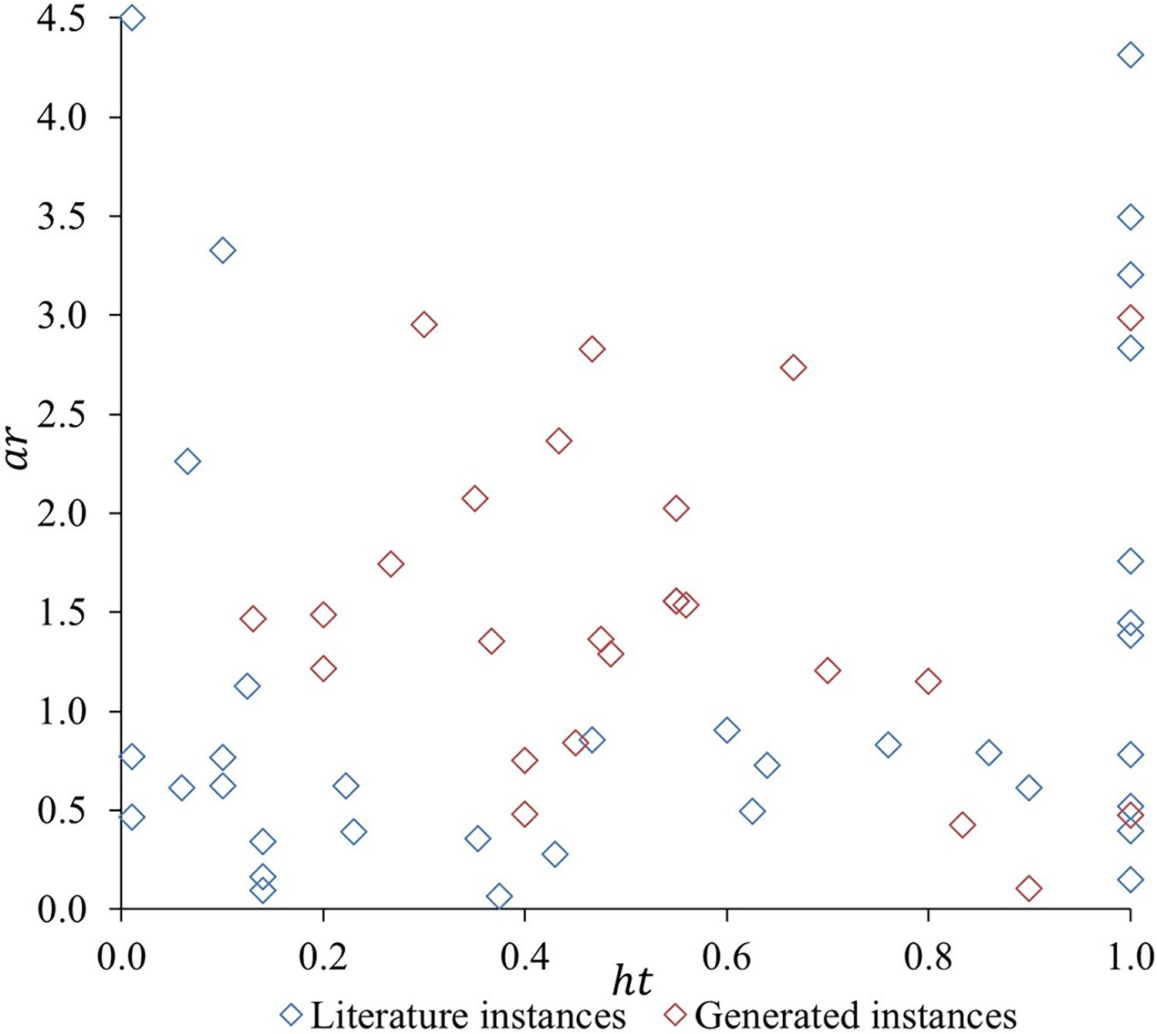
Instances’ distribution considering *ar* and *ht*.

For *ht* and *ar*, the range values were created based on the literature instances considered, generating a uniform instance distribution. Therefore, the *ht* ranges used are low (0≤*ht*≤0.33), medium (0.33<*ht*≤0.66), and high (0.66<*ht*≤1.00). Similarly, the *ar* ranges are low *ar* (0≤*ar*≤0.50), medium *ar* (0.50≤*ar*≤1.00), high *ar* (1.00≤*ar*≤2.00), and very high *ar* (2.00<*ar*). A total of 12 scenarios, composed of five instances, were defined by the combination of different *ar* and *ht* values. Fig 4 shows the distribution considering the scenarios’ characteristics.

From the lack of specific instances approaching sheet metal plasma cutting operations considering real-life constraints, literature instances were used. The literature instances were established on practical operations found in different industries, including steel, wood, and glass cutting [34–39]. Thus, the results obtained can be utilized to find tendencies related to raw material waste considering the sheet metal plasma cutting context.

Therefore, 36 instances were selected from *bwmv* [35], *C* [36], *cgcut* [37], *gcut* [38], *ngcut* [39], *path* [34], and *pt* [40]. The characteristics and corresponding scenarios of each instance are detailed in Table B.1, located in S2 Appendix. In specific scenarios (3, 4, 5, 6, 7, 8, 9, 11, and 12) an adequate number of instances are not available in the literature. Consequently, to ensure a comprehensive dataset for analysis, a total of 24 additional instances (*fran*) were developed.

The new instances were specifically designed to fulfill the scenarios’ requirements without suitable literature instances using the *ar* and *ht* values. Also, the new instances are derived from existing literature instances variations. Thus, the *n* and the rectangles’ dimensions were randomly modified for each problem instance, considering the *ht* values from the required scenarios as reference. In addition, the *W* was determined based on the *ar* values from the necessary scenarios. Also, the new set of instances (*fran*) are included in the supplementary materials. For the remaining sections, in addition to aspectratio, heterogeneity, and number of rectangles, the unit of measurement is defined as “units”.

An indicator named *waste* is used to verify the packing arrangement condition, given by the difference between *H* and *ML*_0_ in Eq (20). Furthermore, higher *waste* values are related to worse packing arrangement, based on empty spaces found between rectangles (apart from *β* and *δ*), resulting in a higher sheet metal waste. Similarly, for lower *waste* values, fewer empty spaces can be verified, and the packing arrangement is closer to the *ML*_0_, resulting in low sheet metal waste. For example, considering *W* = 10, *β* = *δ* = 1, no rotation allowed, and all rectangles have *w*_*i*_ = 7 and *h*_*i*_ = 1, concerning the *x*-axis, is impossible for the rectangles to be allocated side by side, originating an empty space with an area value of 1 unit for each rectangle.


$$waste=\left[\frac{\left(H-ML_{0}\right)}{ML_{0}}\right]100$$ (20)

All packing arrangements were classified into five groups: very good (*waste*≤5%), good (5%<*waste*≤10%), medium (10%<*waste*≤20%), bad (20%<*waste*≤30%), and extremely bad (*waste*≥30%). Also, to differentiate between the PCC variations approached, for *β* = *δ* = 1, *β* = *δ* = 2, and *β* = *δ* = 3, the *waste* indicator was represented by *waste*_1_, *waste*_2_, and *waste*_3_, respectively.

### PCC impact for *β* = *δ* = 1

The commercial solver used was CPLEX Studio IDE 20.1.0. No modifications were made regarding parameters. Therefore, the commercial solver was used with default settings and a time limit based on *n*. For instances with *n*≤15, the time limit was 780 seconds, for 15<*n*≤25, 960 seconds, for 25<*n*≤35, 1140 seconds, for 35<*n*≤40, 1320 seconds, and for 40<*n*≤50, 1500 seconds. The computer processor is an Intel® Core™ i5-7200U with 2.5 gigahertz (Quadcore) CPU and 8 gigabyte RAM, running the Windows 10 64-bit operational system.

In eight instances (*fran84*, *fran92*, *gcut1*, *gcut5*, *pt1_23_52*, *pt8_1_90*, *ngcut4*, and *ngcut7*), the optimality of the solution was proven, averaging a running time of 109 seconds. For the remaining instances, where the time limit was exceeded, the optimality was not proven, and the average gap found by CPLEX was 4.97%. Also, the gap was not used to define the packing arrangement because of the imprecision in the gap for instances where the optimality was not proven, returning values of *waste* which are not representative of the packing arrangement originated. The solutions obtained for all instances are shown in S2 Appendix (Table B.2).

**Fig 5:**
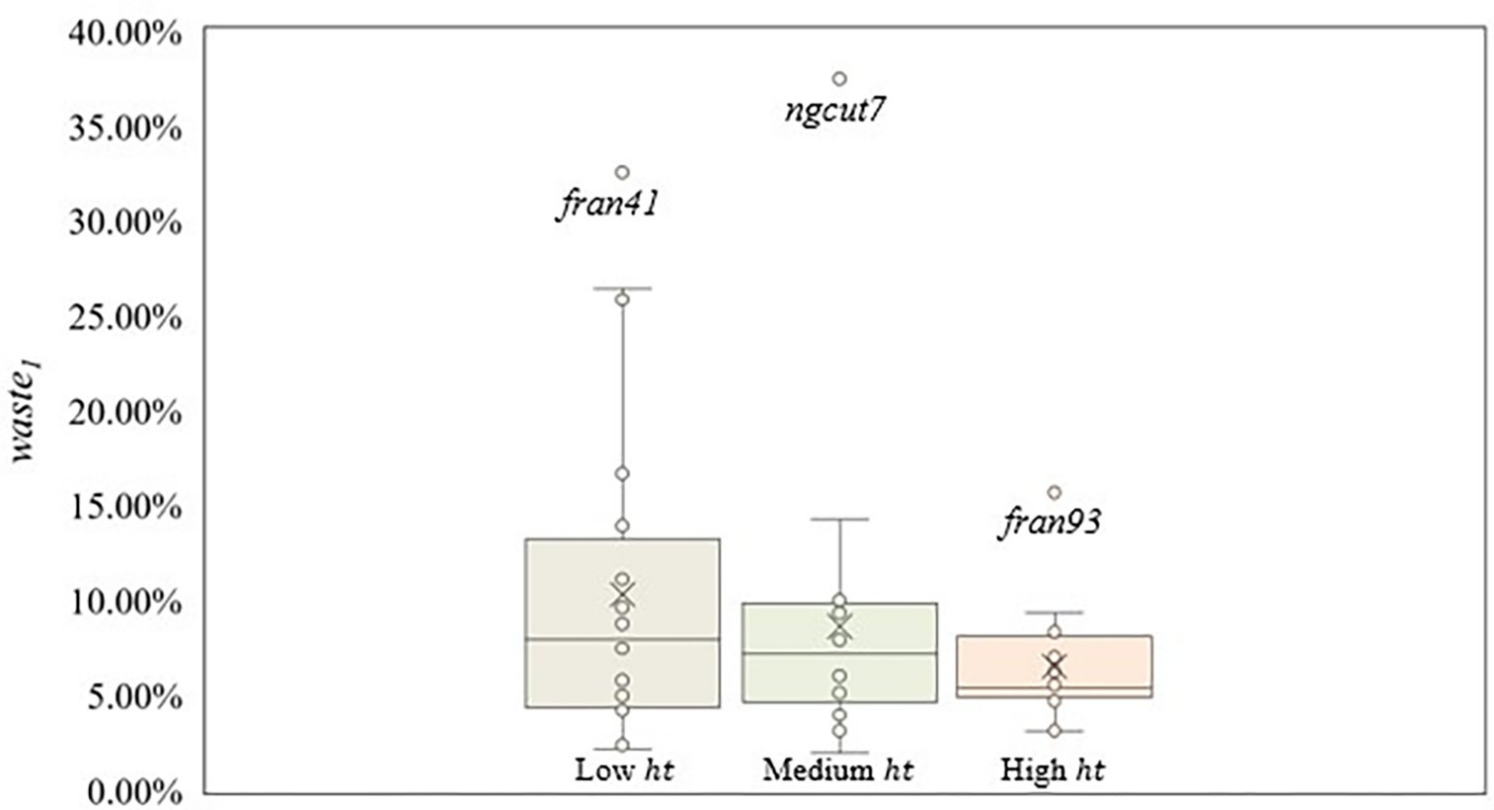
Box plot for *ht* and *waste*_1_ values.

The best solutions were obtained with the scenario considering low *ht* and low *ar*. Therefore, instances from scenario 1 presented the best packing arrangements, with an average *waste*_1_ of 4.37%. For the remaining scenarios, the average *waste*_1_ was 5% to 10%, except for scenario 4 (*waste*_1_ = 21.92%), where the combination of very high *ar* and low *ht* returned bad packing arrangements. Fig 5 shows the box plot considering the *ht* ranges and *waste*_1_ values.

Scenarios with low *ht* (1–4) showed a higher dispersion compared to medium (5–8) and high (9–12) *ht* scenarios, due to scenario 4, where the worst packing arrangements were found, with an average *waste*_1_ of 21.92%, and due to scenario 1, where the best packing arrangements were found, with an average *waste*_1_ of 4.37%. Based on the whiskers’ size, solutions with bad packing arrangements are distant from the average *waste*_1_ (*waste*_1_ = 10.32%). Furthermore, 70.00% of instances showed solutions below the average *waste*_1_, where a new instance with low *ht* has a higher chance of returning less raw material waste.

For the medium *ht* range, the dispersion is lower when compared to the low *ht* range. The scenarios with medium *ht* have similar average *waste*, the highest being scenario 5, with 10.87%, and the lowest being scenario 6, with 6.94%, justifying the dispersion. Apart from the outlier, the distribution of the instances’ packing arrangement is symmetric, as seen by the position of the median. However, 65.00% of instances showed solutions below the average *waste*_1_ (*waste*_1_ = 8.63%), due to the outlier, which raises the average *waste*_1_, following the same pattern encountered in the low *ht* range. Considering only the *ht* variations, instances with a high *ht* returned the best packing arrangements, with the lowest average *waste*_1_ (*waste*_1_ = 6.51%) and dispersion. Also, three instances were considered outliers, returning an uncommon *waste*_1_ value when compared to instances from the same *ht* range. Outliers have a combination of characteristics, discussed at the end of Section 3.2, that result in elevated raw material waste.

**Fig 6:**
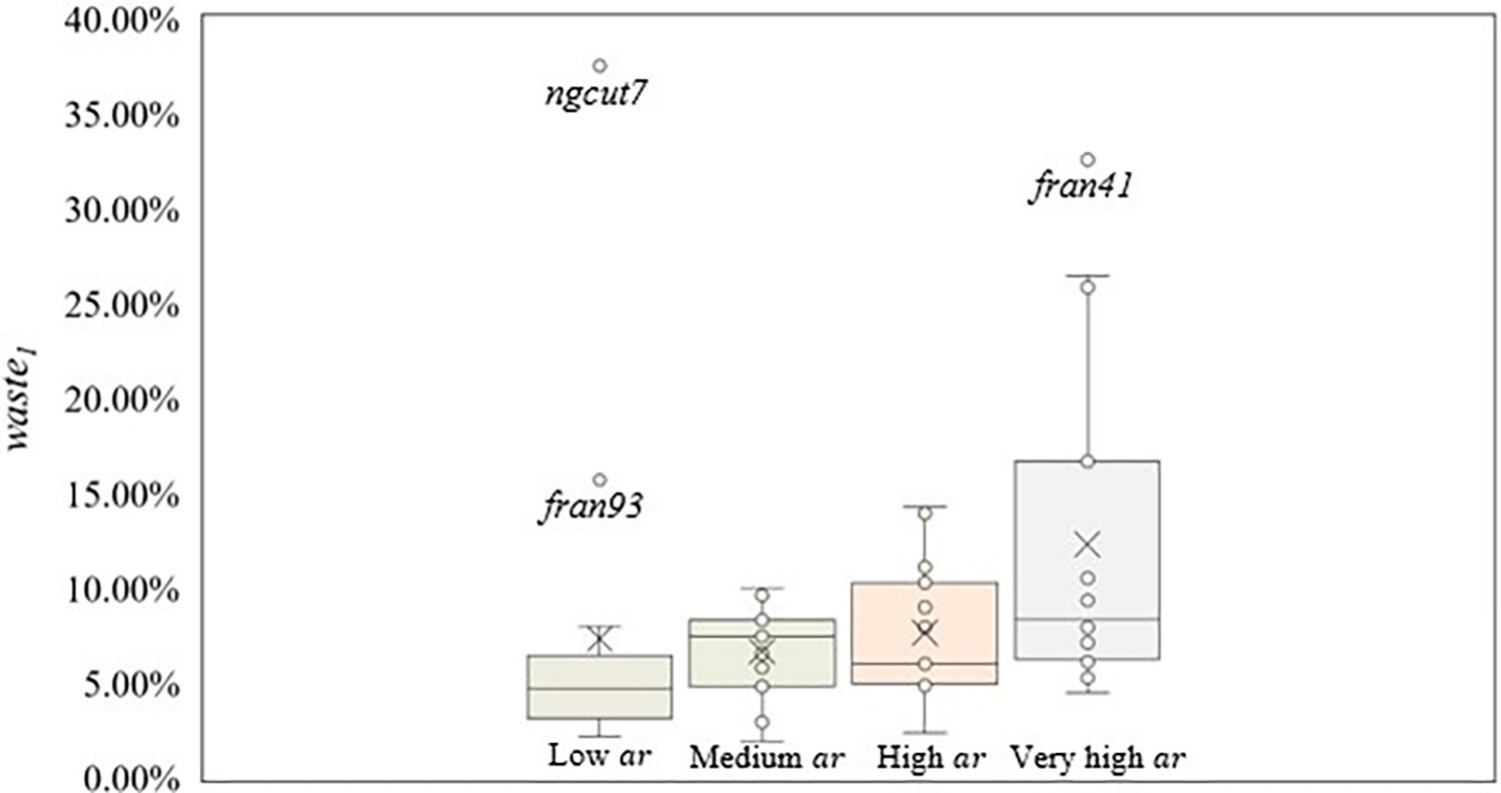
Box plot for *ar* ranges used and the *waste*_1_ values.

Next, Fig 6 shows the box plot considering the *ar* ranges used and *waste*_1_ values. The highest dispersion was found in scenarios with very high *ar* (4, 8, and 12), due to the presence of scenario 4, composed of instances with the worst packing arrangements (average *waste*_1_ = 21.92%), and scenario 12 (average *waste*_1_ = 7.27%). In addition, considering the average *waste*_1_ for scenarios with very high *ar* (*waste*_1_ = 12.29%), 73.30% of instances showed solutions below the average *waste*_1_, due to *fran41* being an outlier, raising the average *waste*_1_ value.

Scenarios with high *ar* (3, 7, and 11) showed a higher dispersion compared to scenarios with low and medium *ar*, despite having similar values for the average *waste*_1_ found. Considering scenarios with low *ar* (1, 5, and 9) and medium *ar* (2, 6, and 10), both have similar dispersion. However, due to scenario 1 (*waste*_1_ = 4.37%), the dispersion range from scenarios with low *ar* is lower, returning instances with very good packing arrangements. Instances with low *ar* should be prioritized due to the dispersion found, despite the higher average *waste*_1_ than scenarios with medium *ar*, presenting solutions with better packing arrangement.

**Fig 7:**
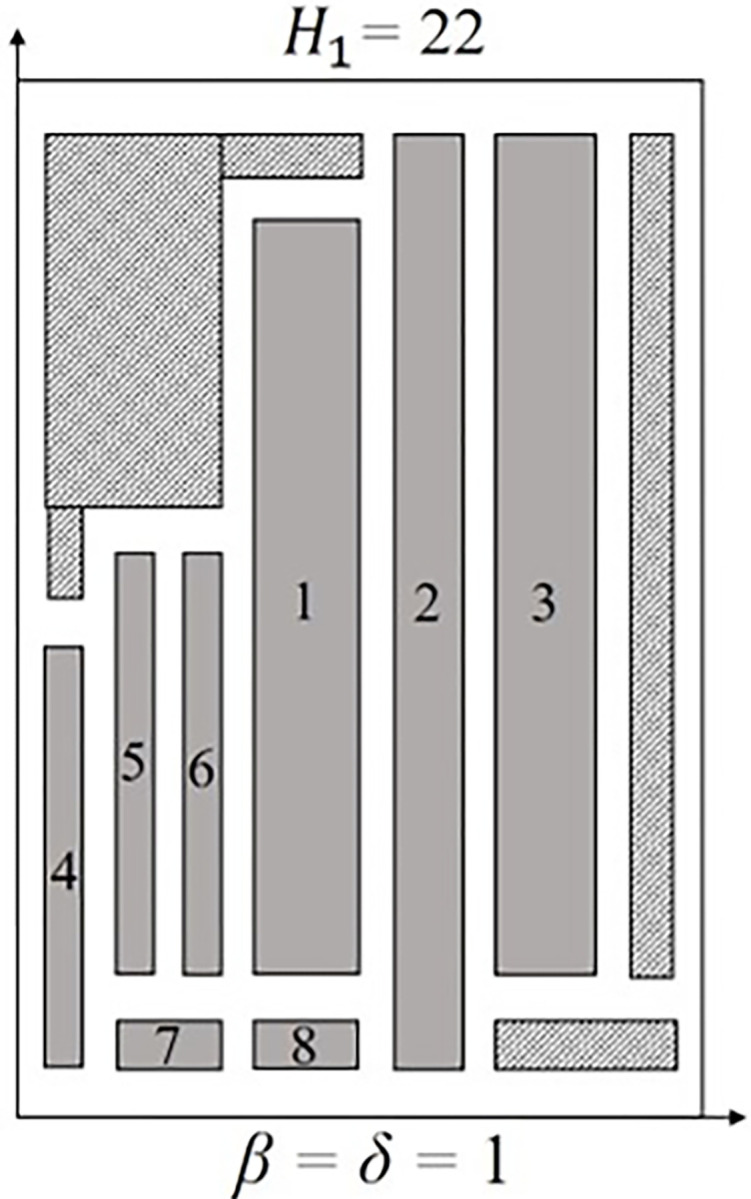
Packing arrangement for ngcut7 with *β* = *δ* = 1. The blank area represents the space occupied by *β* and *δ*, while the hatched area represents the empty spaces formed.

Three instances are outliers (*fran41*, *fran93*, and *ngcut7*). For *fran41* and *ngcut7*, the combination of rectangles dimension, *n*, and *W*, had the most impact on the packing arrangement. Instances with relatively large rectangles, compared to *W*, showed the worst packing arrangement, as seen in *ngcut7* (*waste*_1_ = 37.50%) and *fran41* (*waste*_1_ = 32.56%). The larger the rectangles are compared to *W*, the harder is to position the rectangles, which may develop empty spaces, resulting in sheet metal waste. However, depending on the size of the remaining rectangles, the empty spaces formed can be filled or not, influencing the packing arrangement. Also, *fran93* is considered an outlier only due to being grouped with instances that have better packing arrangements, not presenting the characteristics seen in *fran41* and *ngcut7*. Fig 7 shows the *ngcut7* packing arrangement.

The orientation constraint can reduce the impact related to the combination of *W* and rectangles size, allowing the rectangle to be rotated. Without the orientation constraint and considering the PCC applied, a feasible solution for *ngcut7* would not be possible. Rectangles 1, 2, and 3 are relatively large compared to *W*, which leads to a limited number of suitable positions, helping the formation of empty spaces. Furthermore, it is unfeasible to rotate rectangle 2, causing *H*_1_ to be at least 22 units. The rectangle 2 rotation impossibility forces rectangle 1 and rectangle 3 to not be rotated, occupying 40% of *W*. A total of 72 units of area are empty spaces that could be filled with different rectangles. In addition, rotating and alternating the positions of the remaining rectangles do not reduce the sheet metal wasted.

In summary, scenarios with a low *ht* and a low *ar* provided the best solutions, while a combination of very high *ar* and low *ht* returned the worst packing arrangements. Low and medium *ar* ranges tend to produce good packing arrangements. However, due to the dispersion, low *ar* instances should be prioritized. Also, instances with high *ht* showed less dispersion and a better packing arrangement. For *n*, instances with 30<*n*≤50 produced the worst solutions, which can be related to the difficulty in finding different layout combinations due to the elevated *n*. Good packing arrangements were found in instances with 10<*n*≤30, being attributed to the relatively high number of possible rectangles combinations, returning improved layouts concerning empty spaces. However, instances with *n*≤10 returned medium packing arrangements, therefore, *n* alone should not be used to evaluate the packing arrangement quality. The combination of rectangles dimensions, *n*, and *W*, showed the most impact on the packing arrangement, where the larger the rectangles, the harder is to find good packing positions, leading to the creation of empty spaces.

### Equal increase in the plasma cutting constraints (*β* = *δ* = 2 and *β* = *δ* = 3)

Factors related to the sheet metal cutting operation can affect the PCC value. The plasma machine cutting nozzle may have different diameters, directly impacting in *β*, where higher cutting nozzle diameters result in higher *β* values. Also, during the cutting process, the sheet metal can dislocate due to residual stress, generating scratches, compromising the rectangle’s visual appearance and functionality, requiring different *β* values based on the estimated dislocation. Furthermore, the sheet metal edges are highly irregular due to material transportation, incorrect storage, and mishandling before and during the cutting process, which justifies the possibility of different *δ* values. Therefore, in this section, an equal increase in *β* and *δ* is proposed. The main goal is to verify how an equal increase in PCC affects the packing arrangement by analyzing the *waste* values found.

This section approaches the results from *β* = *δ* = 2 and *β* = *δ* = 3. The optimality was proven in 8 instances (*fran84*, *fran92*, *gcut1*, *gcut5*, *pt1_23_52*, *pt8_1_90*, *ngcut4*, and *ngcut7*), averaging a running time of 95 seconds for *β* = *δ* = 2 and 65 seconds for *β* = *δ* = 3, being highlighted in S2 Appendix (Table B.3). Also, instances without optimality proven showed an average gap by CPLEX of 5.12% and 5.37%, for *β* = *δ* = 2 and *β* = *δ* = 3, respectively.

**Fig 8:**
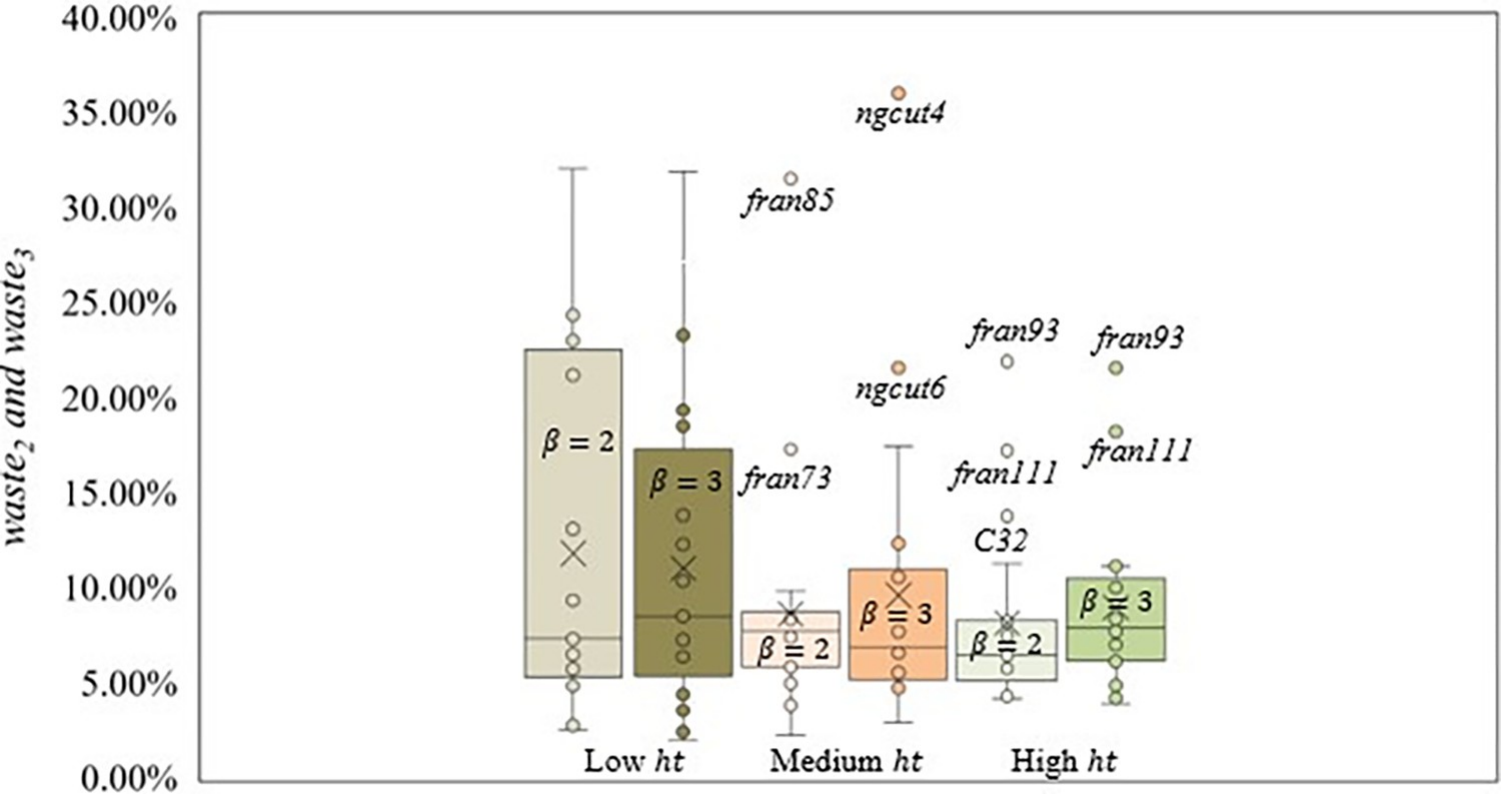
Box plot comparing *waste* values for *β* = *δ* = 2 and *β* = *δ* = 3 considering *ht* ranges.

**Fig 9:**
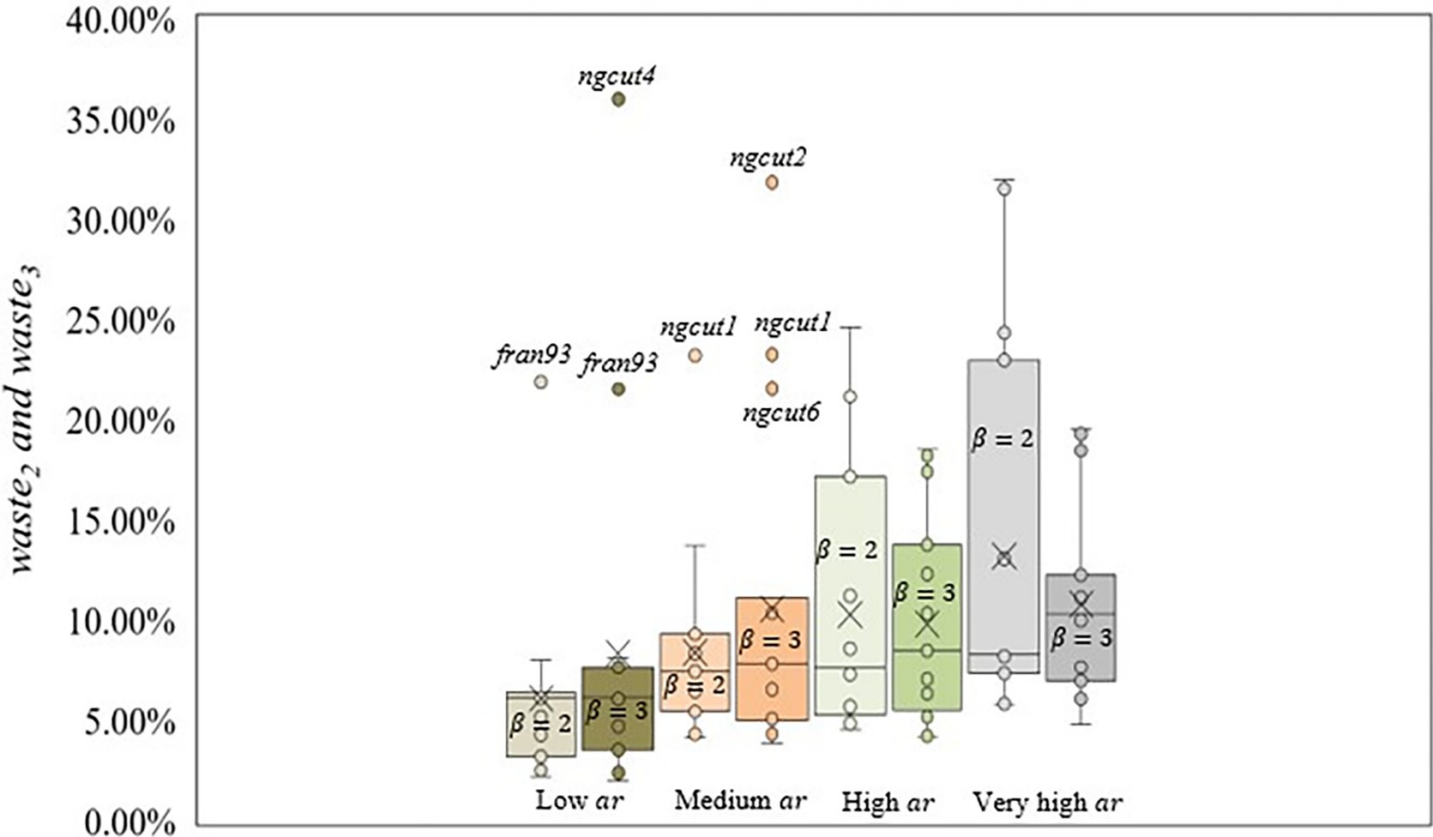
Box plot comparing *waste* values for *β* = *δ* = 2 and *β* = *δ* = 3 considering *ar* ranges.

For *β* = *δ* = 2, scenario 4, with very high *ar* and low *ht* presented the worst packing arrangements, with an average *waste*_2_ of 19.93%, while scenarios 1 and 5, with low *ht* and low and medium *ar* provided the best average solutions, 3.97% and 5.29%, respectively. Also, for *β* = *δ* = 3, scenario 1, with low *ht* and low *ar*, returned the best average solutions (*waste*_3_ = 4.11%), and scenario 4, with high *ar* and low *ht*, returned the worst packing arrangements (*waste*_3_ = 15.24%). In Fig 8, the box plot considering the *ht* ranges and *waste* values for *β* = *δ* = 2 and *β* = *δ* = 3 is presented and Fig 9 shows the box plot for the *ar* ranges and *waste* values for *β* = *δ* = 2 and *β* = *δ* = 3.

The scenarios with medium (5–8) and high (9–12) *ht* followed the pattern found in *β* = *δ* = 1, decreasing the dispersion as the *ht* ranges increased. For *β* = *δ* = 2, scenarios with medium and high *ht* presented similar dispersion values. However, the dispersion in scenarios with high *ht* is better located, returning solutions with less raw material waste. Also, the dispersion found for *β* = *δ* = 3 shows the worst solutions as the PCC increases. Considering scenarios with low *ht* (1–4), the dispersion in *β* = *δ* = 2 was worse than the dispersion for *β* = *δ* = 3 due to instances (*cgcut1*, *fran31*, and *fran41*) returning better solutions with the increase in the PCC from *β* = *δ* = 2 to *β* = *δ* = 3, minimizing the negative impact of scenario 4 in the dispersion found. Scenarios with high *ht* values showed the lowest average *waste*, 8.10% and 9.05%, respectively, followed by scenarios with medium and low *ht*.

Scenarios with low *ar* (1,5, and 9) presented the lowest average *waste* value. For *β* = *δ* = 2, considering the dispersion, with the *ar* increase, the *waste* also increases. When comparing scenarios with high (3, 7, and 11) and very high *ar* (4, 8, and 12), the dispersion for *β* = *δ* = 2 is higher than for *β* = *δ* = 3, due to instances such as *cgcut1*, *fran31*, *fran41*, and *fran85*, presenting an increase or similar *waste* from *β* = *δ* = 1 to *β* = *δ* = 2, followed by a decrease from *β* = *δ* = 2 to *β* = *δ* = 3.

A total of nine instances (*C32*, *fran73*, *fran85*, *fran93*, *fran111*, *ngcut1*, *ngcut2*, *ngcut4*, and *ngcut6*) are outliers, as seen in Figs 8 and 9, presenting a combination of characteristics that impacted the *waste*. However, *fran73* does not have the same characteristics, being grouped with better packing arrangement instances, and not having a specific reason for being an outlier.

**Fig 10:**
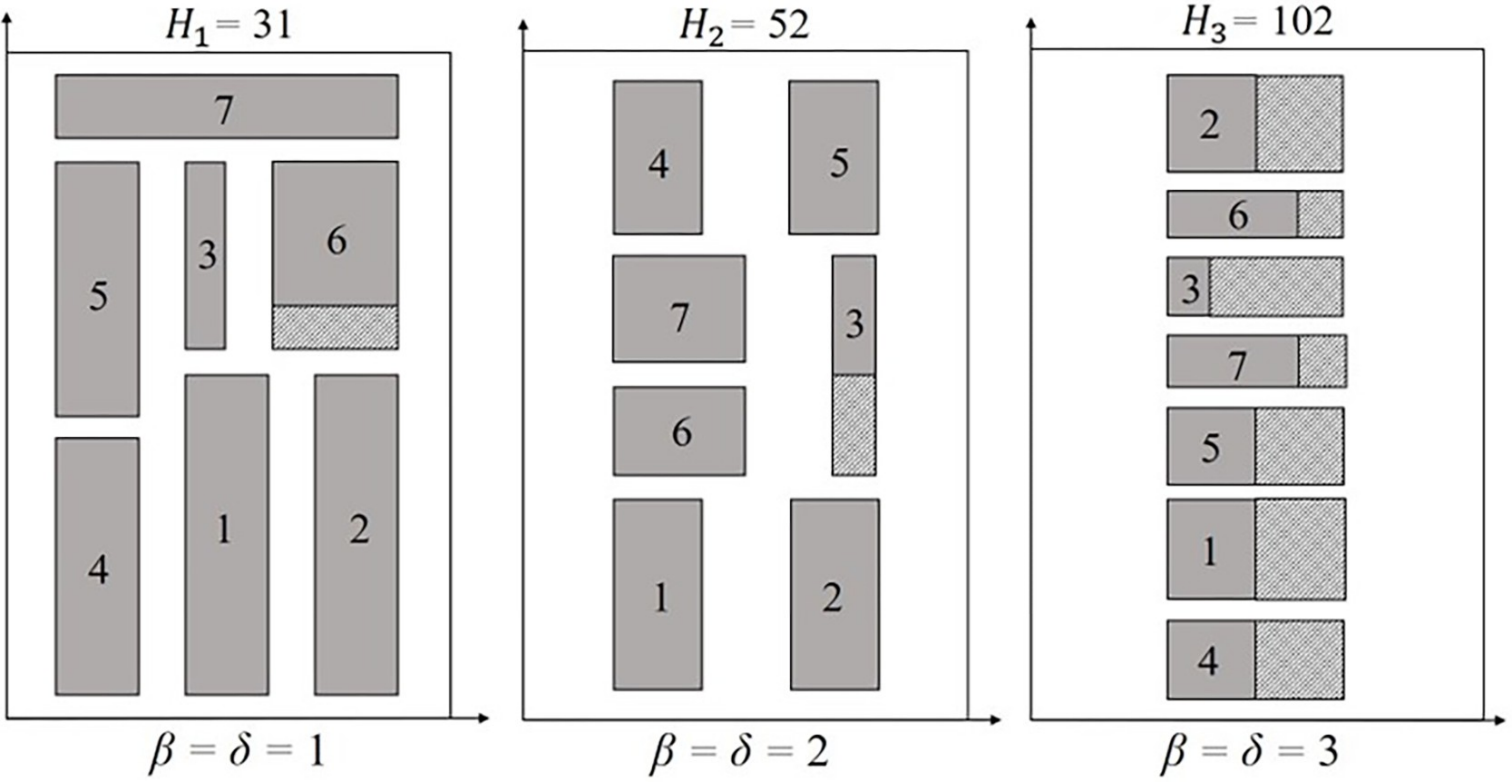
Packing arrangement for ngcut4 for *β* = *δ* = 1, *β* = *δ* = *2*, and *β* = *δ* = 3. The *y*-axis values were proportionally adjusted based on *H*_1_, being the packing arrangement from *H*_2_ multiplied by 0.59 and the packing arrangement from *H*_3_ by 0.30.

With *β* = *δ* = 2, the relation between rectangles dimensions and strip size increases, where packing arrangements with smaller rectangles may develop empty spaces. The impact can be seen in smaller width instances, such as *fran85*, where the increase in PCC significantly aggravated the packing arrangement (*waste*_1_ = 6.10% to *waste*_2_ = 31.50%). As expected, the same pattern is found for *β* = *δ* = 3, seen in instances such as *ngcut4*, where *waste* increases from *β* = *δ* = 2 to *β* = *δ* = 3 (*waste*_2_ = 6.12% to *waste*_3_ = 36.00%). Fig 10 shows the *ngcut4* packing arrangement.

An empty space (*waste*_1_ = 3.33%) was obtained considering *β* = *δ* = 1. A slightly large empty space (*waste*_2_ = 6.12%) was verified for *β* = *δ* = 2. Finally, with *β* = *δ* = 3, the rectangles were forced to be packed one by level due to *W* = 10, creating more empty spaces (*waste*_3_ = 36.00%), impacting the sheet metal waste. Instances such as *C32*, *cgcut1*, *fran85*, *fran93*, *fran111*, *fran112*, *ngcut1*, *ngcut2*, *ngcut4*, and *ngcut6* followed the same pattern.

**Fig 11:**
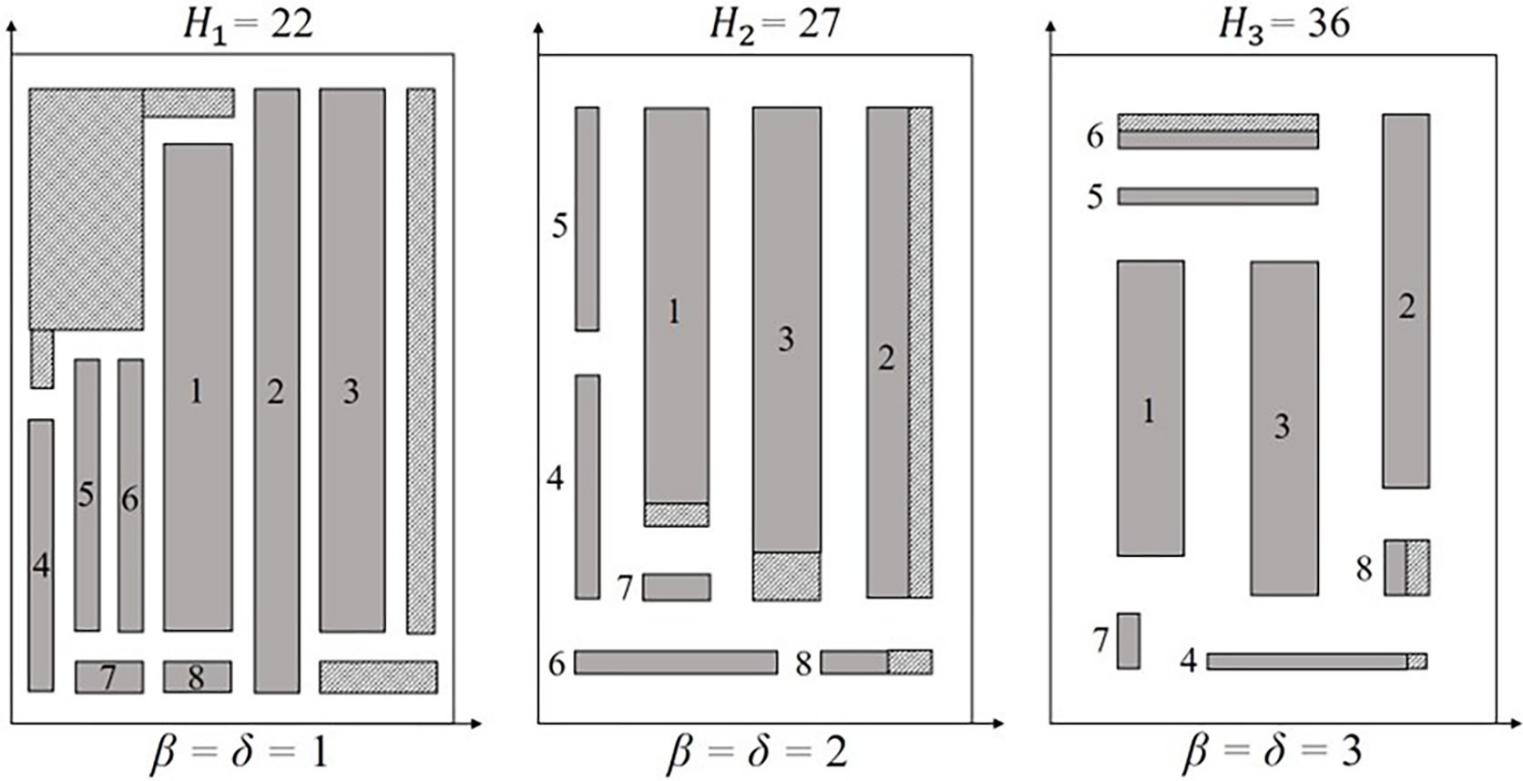
Packing arrangement for ngcut7 for *β* = *δ* = 1, *β* = *δ* = 2, and *β* = *δ* = 3. The *y*-axis values were proportionally adjusted based on *H*_1_, being the packing arrangement from *H*_2_ multiplied by 0.82 and the packing arrangement from *H*_3_ by 0.61.

Furthermore, instances such as *cgcut1*, *fran41*, *fran31*, and *fran85* presented a better packing arrangement with the PCC increase. The main reason is related to the way the rectangles fit with the PCC values, which can lead to improved solutions. For *β* = *δ* = 1 to *β* = *δ* = 2, the best improvement was shown by *ngcut7*, varying from *waste*_1_ = 37.50% to *waste*_2_ = 8.00%. Considering *β* = *δ* = 2 to *β* = *δ* = 3, *fran85* improved the most, varying from *waste*_2_ = 31.50% to *waste*_3_ = 7.85%. Fig 11 shows the packing arrangement for *ngcut7*.

A high number of empty spaces (*waste*_1_ = 37.50%) was found in the *ngcut7* packing arrangement for *β* = *δ* = 1, based on the rectangles size, *n*, lack of rectangles with adequate dimensions to minimize empty spaces, and how the rectangles were impacted by the PCC. However, for *β* = *δ* = 2, a packing arrangement with less sheet metal waste (*waste*_2_ = 8.00%) was obtained. Finally, *β* = *δ* = 3 originates an improved layout, minimizing empty spaces and improving the packing arrangement (*waste*_3_ = 2.86%). The improvement in the packing arrangement, as the PCC increases, is due to the presence of larger rectangles compared to *W*, as well as smaller rectangles, which cannot fill the empty spaces originating from the larger rectangles’ positioning. As *β* and *δ* increase, the empty spaces are filled with smaller rectangles and the PCC, reducing sheet metal waste. Instances with the same behavior, especially from*β* = *δ* = 2 to *β* = *δ* = 3, are *cgcut1*, *fran41*, *fran31*, and *fran85*.

For *β* = *δ* = 2 and *β* = *δ* = 3, scenarios with low *ht* and low *ar* showed the best packing arrangement, while scenarios with low *ht* and very high *ar*, returned the worst packing arrangement. Generally, the increase in *β* and *δ* negatively affected the *waste* values found. Instances with high *ht* presented better packing arrangements. Also, instances with low *ar* showed the best average *waste* values found. Instances with 30<*n*≤50 produced the worst packing arrangements for *β* = *δ* = 2, while instances with *n*≤30 returned good and medium packing arrangements. However, for *β* = *δ* = 3, no clear pattern regarding the *n* and the *waste*_3_ was found, since instances with *n*≤10 produced the worst packing arrangements, instances with a high *n* (30<*n*≤50) returned medium packing arrangements, and instances with a medium *n* (20<*n*≤30) returned good packing arrangements. With the increase in the PCC, the relation between rectangles dimension and strip size also increases, specifically in smaller width instances. However, in five instances, the increase in the PCC led to a significant improvement in the packing arrangement.

## Summary

A summary of the main findings obtained is presented. First, a discussion related to the *ML*_0_ precision, considering variations in the PCC, was developed. Second, a relation between the problem variables and the *waste* obtained is analyzed. Third, tendencies found in the instances’ behavior due to the PCC, regarding sheet metal waste, are presented.

The *ML*_0_ precision is calculated with optimal solutions. Therefore, instances where an optimal packing arrangement was found (*fran84*, *fran92*, *pt1_23_52*, *ngcut4*, and *ngcut7*), considering all PCC variations, were used to evaluate the *ML*_0_. The average difference for the *ML*_0_ solution, which refers to a packing without empty spaces, apart from the PCC, and the optimal solution, was 14.26%. Therefore, the *ML*_0_ can be used to compare and evaluate the solutions found. In addition, *β* = *δ* = 2 presented the most accurate *ML*_0_, averaging a 10.71% difference. For *β* = *δ* = 1, the average difference found was 16.55%, and for *β* = *δ* = 3, the average difference was 15.51%, showing the *ML*_0_ imprecision does not have a direct relation with the PCC increase.

No clear relation was found between only one problem variable and the *waste* obtained. The 2D-SPP variation approached is multicriterial, where only one variable is insufficient to find tendencies related to raw material waste. Therefore, for the 2D-SPP, the combination of the strip size, *n*, rectangles dimensions, and the PCC values, was the main factor impacting the solution packing arrangement and the *waste* obtained, as well as the number of empty spaces. Furthermore, the 2D-SPP is classified as an NP-hard problem, being impossible to find a linear relation between the problem characteristics and the packing arrangement obtained. However, tendencies can be determined based on the results found.

The increase in *waste* from *β* = *δ* = 1 to *β* = *δ* = 3 is a result of the combination between *W*, *n*, PCC values, and rectangles dimensions. For smaller instances (*W*≤10), as the PCC values increase, especially from *β* = *δ* = 2 to *β* = *δ* = 3, fewer rectangles are packed in each level, increasing the *waste* obtained. For example, the seven rectangles of *ngcut4* cannot be rotated during the packing process, due to one rectangle dimension being relatively large compared to *W* (*W* = 10). In addition, the proportion of strip occupied by empty spaces increases as the PCC values rise, being 0.98% for *β* = *δ* = 1 and 17.24% for *β* = *δ* = 3. The worse *waste* was found where considering the *x*-axis and *W* = 10, only 4 units can be effectively used to pack rectangles, due to *δ* = 3. The generated packing arrangement contains only one rectangle per level, as verified in Fig 10, severely increasing the *waste* and the *ML*_0_ imprecision. Instances such as *C32*, *cgcut1*, *fran31*, *fran75*, *fran85*, *fran93*, *fran111*, *fran112*, *ngcut1*, *ngcut2*, and *ngcut6*, presented the same pattern, with an increase in the *waste* as the PCC values escalated.

Instances with *W*≥10 can also be affected by the PCC increase, depending on the rectangles’ geometric dimensions. Proportionally large rectangles compared to *W*, combined with higher PCC values, can originate a packing arrangement with only one rectangle per level, increasing the *waste*. From the instances analyzed and the PCC values proposed, smaller width instances showed a high increase in *waste* derivate from the packing arrangement. Instances with a larger *W* generally had a lower *waste*. As the strip size increases, more possibilities for the rectangles to be rearranged are created to avoid the need to pack fewer rectangles per level, decreasing the *waste* found and the sheet metal waste.

Another behavior was found where, as the PCC values increase, the *waste* encountered was lower. Instances with larger and smaller rectangles compared to the *W* can generate more empty spaces, due to the number of smaller rectangles not being enough to fill the empty spaces created by the larger rectangles. With increasing PCC, the empty spaces are filled by small rectangles and the new constraint values, reducing waste. However, eventually, with the continuous increase in the PCC, fewer rectangles will be packed by level, increasing the *waste*. For example, in *ngcut7*, three rectangles cannot be rotated, being relatively larger compared to *W*, and 5 rectangles are relatively small compared to *W*. The larger rectangles increase the height obtained and consequently the strip area. Since the remaining rectangles are not enough to fulfill the generated area, empty spaces are formed. However, with the increase in the PCC, the empty spaces began to be filled, as verified in Fig 11. Also, the proportion of empty spaces for *β* = *δ* = 1 is 30.95% and 1.12% for *β* = *δ* = 3. The behavior was seen in *fran31*, *fran41*, *fran85*, *pt1_23_52*, and *pt5_23_52*.

Larger values for the PCC are unfeasible in practical applications, specifically in smaller instances (*W*≤10), returning a packing arrangement where only one rectangle can be packed by level, leading to a higher amount of material waste. In addition, the PCC must respect the minimum distance related to the plasma cutting machine used, where adjacent rectangles are not melted by the plasma torch heat, being the minimum distance directly related to the cutting nozzle size and the sheet metal thickness.

## Conclusion

We verified the impact of the PCC in the sheet metal waste context by analyzing the packing arrangement obtained in well-known and generated rectangular 2D-SPP instances. For sheet metal plasma cutting, two practical constraints were approached, the minimum distance between the rectangles and the minimum distance between the rectangles and the strip edges. First, an equal increase in the PCC was proposed. Second, an individual increase in both PCC was explored.

A modified lower bound (*ML*_0_) was used to compare and evaluate the 2D-SPP solutions, considering the PCC. Furthermore, the average difference between *ML*_0_ and the solutions with proven optimality is 14.26%. The reason for *ML*_0_ inaccuracy was related to the empty spaces, which are not considered in *ML*_0_ calculation. However, the *ML*_0_ can be used as a reference value to compare and evaluate the solutions obtained, especially since there is a lack of studies approaching PCC in the 2D-SPP academic scenario for comparison purposes.

To evaluate the PCC impact, 36 instances from the literature were selected and 24 instances were generated, being organized in 12 scenarios, based on variations in *ht* and *ar*. For *β* = *δ* = 1, the best packing arrangements were found in scenarios with a low *ht* and a low *ar*, and the worst packing arrangements were found in scenarios with very high *ar* and low *ht*. Considering *β* = *δ* = 2 and *β* = *δ* = 3, the same pattern for *β* = *δ* = 1 was encountered, where scenarios with low *ht* and low *ar* showed the best packing arrangements, and scenarios with low *ht* and very high *ar*, returned the worst packing arrangements.

The main factor impacting the packing arrangement due to the PCC was a combination of rectangles size, *n*, and *W*, due to the difficulty in positioning rectangles, generating empty spaces. Overall, the relation between rectangles dimension and strip size increases with higher PCC values, specifically for smaller width instances, affecting the packing arrangement severely. However, for 28 instances, the increase in *β* and *δ* originate a better layout, improving the packing arrangement.

In the sheet metal waste context, due to the PCC, instances with *W*≤10 should be avoided in practical operations, regardless of the scenario, as smaller width instances returned the worst packing arrangement for *β* = *δ* = 2 and *β* = *δ* = 3. Considering *β* = *δ* = 1, smaller width instances can be used in practical operations, as the packing arrangement found was acceptable. However, the relation of rectangles size and *W* should be analyzed, as instances with larger rectangles compared to *W* originated the worst solutions. To maximize the sheet metal usage, instances with low *ar* or low *ht* should be prioritized, since better packing arrangements were obtained across all PCC variations.

A gap was found in the 2D-SPP literature regarding constraints from real-life scenarios, apart from the orientation and guillotine cutting constraint. This research helps fill the gap, presenting the impact of practical constraints in the rectangular 2D-SPP context. Considering the academic scenario related to the 2D-SPP research, this is the first article to address the specific constraints imposed by the sheet metal plasma cutting process, presenting different packing layouts when compared to 2D-SPP practical articles, due to the PCC. The robustness of the results is supported using a comprehensive set of instances and scenarios, highlighting the potential to effectively address the waste in sheet metal plasma cutting. By aiding the decision-making process and enhancing operational management, the findings presented in this research offer valuable insights into the practical implications of these real-life constraints on raw material waste reduction.

Furthermore, the present articles’ applicability extends beyond plasma cutting, encompassing other metal sheet cutting processes such as laser cutting, waterjet cutting, and oxyfuel cutting. To adapt the mathematical model, the PCC values must be modified considering the approached operation particularity. Also, constraints involving rectangle position must be analyzed, since a different diagonal distance between rectangles can be necessary to minimize the heat-affect zone effect, as seen in oxyfuel cutting. Finally, additional constraints including heat sensitivity, cut accuracy and tolerance, as well as abrasive material use, can be considered for waterjet cutting. For laser cutting, cutting machine alignment, cut speed, sheet thickness, and heat input, may be included. Lastly, for oxyfuel, cut quality for post-processing operations, and a higher heat-affected zone, can be incorporated in the model.

Future research can focus on the novel solution methods development, particularly heuristics and approximation algorithms, to solve the 2D-SPP with the PCC, addressing instances with a larger number of rectangles and achieving faster solution times. Also, different plasma cutting technical aspects can be explored, verifying if the raw material tendencies found can minimize operational costs, including electric energy, cut time, and gas usage. Furthermore, the packing layout added-value can be analyzed to find a relation between raw material waste and added-value. Finally, different plasma cutting constrains, such as cut pistol trajectory, sheet metal with varying unusable areas, different materials, and different items geometry, can be investigated.

## Supporting information

S1 Text (TXT)

S1 Appendix Basic notation. (DOCX)

S2 Appendix Tables. (DOCX)
