# Supplementary material for: Impact of minimum distance constraints on sheet metal waste for plasma cutting
Source: PLoS One. 2023 Sep 27;18(9):e0292032. doi: 10.1371/journal.pone.0292032 (PMC10529572; doi:10.1371/journal.pone.0292032)
Supplement: S1 Appendix — (DOCX) [file pone.0292032.s002.docx]

# Appendix A: Basic Notation

$\beta$: minimum distance between rectangles;

$\delta$: minimum distance between rectangles and the strip edges;

$I$: set of rectangles;

$i$: rectangle belonging to *I;*

$h_{i}$: rectangle height;

$w_{i}$: rectangle width;

$x_{i}$: rectangle position in the $x$-axis;

$y_{i}$: rectangle position in the $y$-axis;

$\alpha_{i}:$ Boolean variable related to rectangle rotation;

$z_{1ij}, z_{2ij}, z_{3ij}$, and $z_{4ij}:$ Boolean variable used in Big-M method to avoid rectangle overlapping;

$\text{M}$: A large number used in Big-M method to avoid rectangle overlapping;

*H*: strip height solution;

$W$: strip width;

$L_{h}$: first bound related to the tallest rectangle’s height;

$L_{c}$: continuous lower bound;

$L_{o}$: area lower bound;

${ML}_{h}$: modified first bound related to the tallest rectangle height;

${ML}_{c}$: modified continuous lower bound;

${ML}_{o}$: modified area lower bound;

$waste$: difference found for the $H$ and the ${ML}_{o}$;

$D_{1}$: higher strip dimension;

$D_{2}$: lower strip dimension;

$d_{1i}$: higher rectangle dimension;

$d_{2i}$: lower rectangle dimension;

$ar$: aspectratio;

$ht$: heterogeneity;

$n$: number of rectangles;

$nt$: number of different rectangles;

$A1$: area related to the rectangles packed into the strip and the minimum distance constraints;

$A2$: strip bottom area;

$A3$: strip left area.\S
