# Supplementary material for: Impact of minimum distance constraints on sheet metal waste for plasma cutting
Source: PLoS One. 2023 Sep 27;18(9):e0292032. doi: 10.1371/journal.pone.0292032 (PMC10529572; doi:10.1371/journal.pone.0292032)
Supplement: S2 Appendix — (DOCX) [file pone.0292032.s003.docx]

# Appendix B: Tables

**Table B.1 - Characteristics of each instance.**

| **Scenario** | **Instance** | $\boldsymbol{n}$ | $\boldsymbol{W}$ | $\boldsymbol{ht}$ | $\boldsymbol{ar}$ |
| --- | --- | --- | --- | --- | --- |
| 1 | *ngcut8* | 13 | 20 | 0.23 | 0.39 |
|  | *ngcut12* | 22 | 30 | 0.14 | 0.34 |
|  | *pt8_1_12* | 15 | 139 | 0.01 | 0.46 |
|  | *pt8_1_90* | 14 | 422 | 0.14 | 0.16 |
|  | *pt8_22_3* | 14 | 158 | 0.14 | 0.09 |
| 2 | *ngcut1* | 10 | 10 | 0.10 | 0.63 |
|  | *ngcut2* | 17 | 10 | 0.06 | 0.61 |
|  | *ngcut9* | 18 | 20 | 0.22 | 0.63 |
|  | *pt6_24_34* | 25 | 367 | 0.01 | 0.77 |
|  | *pt8_1_51* | 20 | 207 | 0.10 | 0.77 |
| 3 | *cgcut1* | 16 | 10 | 0.13 | 1.13 |
|  | *fran31* | 35 | 180 | 0.20 | 1.49 |
|  | *fran32* | 30 | 50 | 0.27 | 1.74 |
|  | *fran33* | 20 | 400 | 0.20 | 1.22 |
|  | *fran34* | 23 | 120 | 0.13 | 1.47 |
| 4 | *fran41* | 50 | 200 | 0.30 | 2.95 |
|  | *pt1_23_52* | 10 | 216 | 0.10 | 3.33 |
|  | *pt1_23_94* | 30 | 291 | 0.07 | 2.26 |
|  | *pt1_24_15* | 25 | 763 | 0.01 | 4.50 |
|  | *pt5_23_32* | 13 | 488 | 0.15 | 2.75 |
| 5 | *C12* | 17 | 20 | 0.35 | 0.36 |
|  | *C13* | 16 | 20 | 0.63 | 0.50 |
|  | *fran51* | 20 | 250 | 0.40 | 0.48 |
|  | *ngcut4* | 7 | 10 | 0.43 | 0.28 |
|  | *ngcut7* | 8 | 20 | 0.38 | 0.06 |
| 6 | *C22* | 25 | 40 | 0.60 | 0.90 |
|  | *C31* | 28 | 60 | 0.64 | 0.73 |
|  | *fran61* | 20 | 300 | 0.40 | 0.75 |
|  | *fran62* | 40 | 250 | 0.45 | 0.84 |
|  | *ngcut6* | 15 | 10 | 0.47 | 0.86 |
| 7 | *fran71* | 20 | 400 | 0.55 | 1.56 |
|  | *fran72* | 30 | 50 | 0.37 | 1.35 |
|  | *fran73* | 50 | 150 | 0.56 | 1.54 |
|  | *fran74* | 35 | 65 | 0.49 | 1.29 |
|  | *fran75* | 40 | 200 | 0.48 | 1.36 |
| 8 | *fran81* | 20 | 400 | 0.55 | 2.02 |
|  | *fran82* | 30 | 100 | 0.47 | 2.83 |
|  | *fran83* | 30 | 50 | 0.43 | 2.37 |
|  | *fran84* | 9 | 94 | 0.67 | 2.74 |
|  | *fran85* | 20 | 10 | 0.35 | 2.08 |
| 9 | *C11* | 16 | 20 | 1.00 | 0.40 |
|  | *fran91* | 20 | 90 | 1.00 | 0.47 |
|  | *fran92* | 10 | 20 | 0.90 | 0.41 |
|  | *fran93* | 30 | 50 | 0.83 | 0.43 |
|  | *path1* | 25 | 100 | 1.00 | 0.15 |
| 10 | *bwmv155* | 20 | 100 | 1.00 | 0.52 |
|  | *bwmv160* | 20 | 100 | 0.90 | 0.61 |
|  | *C21* | 25 | 40 | 0.76 | 0.83 |
|  | *C32* | 29 | 60 | 0.86 | 0.79 |
|  | *gcut13* | 32 | 3000 | 1.00 | 0.78 |
| 11 | *fran111* | 20 | 10 | 0.80 | 1.15 |
|  | *fran112* | 20 | 10 | 0.70 | 1.21 |
|  | *gcut1* | 10 | 250 | 1.00 | 1.76 |
|  | *gcut5* | 10 | 500 | 1.00 | 1.38 |
|  | *gcut9* | 10 | 1000 | 1.00 | 1.45 |
| 12 | *fran121* | 15 | 225 | 1.00 | 2.99 |
|  | *gcut2* | 20 | 250 | 1.00 | 2.83 |
|  | *gcut6* | 20 | 500 | 1.00 | 3.21 |
|  | *gcut10* | 20 | 1000 | 1.00 | 3.49 |
|  | *gcut11* | 30 | 1000 | 1.00 | 4.32 |

**Table B.2 - Results for** $\boldsymbol{\beta}\boldsymbol{=}\boldsymbol{\delta}\boldsymbol{=}\boldsymbol{1}$**. Solutions with optimality proven are highlighted.**

| **Scenario** | **Instance** | $\boldsymbol{H}_{\boldsymbol{1}}$ | ${\boldsymbol{M}\boldsymbol{L}_{\boldsymbol{0}}}_{\boldsymbol{1}}$ | $\boldsymbol{waste}_{\boldsymbol{1}}$ |
| --- | --- | --- | --- | --- |
| 1 | *ngcut8* | 47 | 46 | 2.17% |
|  | *ngcut12* | 108 | 100 | 8.00% |
|  | *pt8_1_12* | 327 | 312 | 4.81% |
|  | *pt8_1_90* | **149** | 143 | 4.20% |
|  | *pt8_22_3* | 153 | 149 | 2.68% |
|  | average $waste$ | - | - | 4.37% |
| 2 | *ngcut1* | 35 | 34 | 2.94% |
|  | *ngcut2* | 57 | 52 | 9.62% |
|  | *ngcut9* | 73 | 69 | 5.80% |
|  | *pt6_24_34* | 389 | 362 | 7.46% |
|  | *pt8_1_51* | 273 | 251 | 8.76% |
|  | average $waste$ | - | - | 6.92% |
| 3 | *cgcut1* | 43 | 42 | 2.38% |
|  | *fran31* | 50 | 45 | 11.11% |
|  | *fran32* | 190 | 181 | 4.97% |
|  | *fran33* | 163 | 151 | 7.95% |
|  | *fran34* | 49 | 43 | 13.95% |
|  | average $waste$ | - | - | 8.07% |
| 4 | *fran41* | 57 | 43 | 32.56% |
|  | *pt1_23_52* | **43** | 34 | 26.47% |
|  | *pt1_23_94* | 67 | 62 | 8.06% |
|  | *pt1_24_15* | 49 | 42 | 16.67% |
|  | *pt5_23_32* | 73 | 58 | 25.86% |
|  | average $waste$ | - | - | 21.92% |
| 5 | *C12* | 33 | 32 | 3.13% |
|  | *C13* | 33 | 31 | 6.45% |
|  | *fran51* | 236 | 227 | 3.96% |
|  | *ngcut4* | **31** | 30 | 3.33% |
|  | *ngcut7* | **22** | 16 | 37.50% |
|  | average $waste$ | **-** | - | 10.87% |
| 6 | *C22* | 26 | 24 | 8.33% |
|  | *C31* | 44 | 40 | 10.00% |
|  | *fran61* | 212 | 199 | 6.53% |
|  | *fran62* | 354 | 328 | 7.93% |
|  | *ngcut6* | 53 | 52 | 1.92% |
|  | average $waste$ | - | - | 6.94% |
| 7 | *fran71* | 164 | 156 | 5.13% |
|  | *fran72* | 182 | 165 | 10.30% |
|  | *fran73* | 56 | 49 | 14.29% |
|  | *fran74* | 141 | 133 | 6.02% |
|  | *fran75* | 445 | 407 | 9.34% |
|  | average $waste$ | - | - | 9.01% |
| 8 | *fran81* | 164 | 157 | 4.46% |
|  | *fran82* | 477 | 442 | 7.92% |
|  | *fran83* | 232 | 212 | 9.43% |
|  | *fran84* | **21** | 19 | 10.53% |
|  | *fran85* | 87 | 82 | 6.10% |
|  | average $waste$ | - | - | 7.69% |
| 9 | *C11* | 33 | 32 | 3.13% |
|  | *fran91* | 89 | 85 | 4.71% |
|  | *fran92* | **64** | 61 | 4.92% |
|  | *fran93* | 59 | 51 | 15.69% |
|  | *path1* | 120 | 114 | 5.26% |
|  | average $waste$ | - | - | 6.74% |
| 10 | *bwmv155* | 66 | 64 | 3.13% |
|  | *bwmv160* | 87 | 83 | 4.82% |
|  | *C21* | 26 | 24 | 8.33% |
|  | *C32* | 43 | 40 | 7.50% |
|  | *gcut13* | 5124 | 4789 | 7.00% |
|  | average $waste$ | - | - | 6.15% |
| 11 | *fran111* | 64 | 61 | 4.92% |
|  | *fran112* | 63 | 60 | 5.00% |
|  | *gcut1* | **728** | 668 | 8.98% |
|  | *gcut5* | **1164** | 1103 | 5.53% |
|  | *gcut9* | 2133 | 2034 | 4.87% |
|  | average $waste$ | - | - | 5.86% |
| 12 | *fran121* | 1059 | 1006 | 5.27% |
|  | *gcut2* | 1228 | 1123 | 9.35% |
|  | *gcut6* | 2667 | 2490 | 7.11% |
|  | *gcut10* | 5720 | 5383 | 6.26% |
|  | *gcut11* | 7122 | 6572 | 8.37% |
|  | average $waste$ | - | - | 7.46% |

**Table B.3 - Results for** $\boldsymbol{\beta}\boldsymbol{=}\boldsymbol{\delta}\boldsymbol{=}\boldsymbol{2}$ **and** $\boldsymbol{\beta}\boldsymbol{=}\boldsymbol{\delta}\boldsymbol{=}\boldsymbol{3}$**. Solutions with optimality proven are highlighted.**

| **Scenario** | **Instance** | $\boldsymbol{H}_{\boldsymbol{2}}$ | ${\boldsymbol{ML}_{\boldsymbol{0}}}_{\boldsymbol{2}}$ | $\boldsymbol{waste}_{\boldsymbol{2}}$ | $\boldsymbol{H}_{\boldsymbol{3}}$ | ${\boldsymbol{ML}_{\boldsymbol{0}}}_{\boldsymbol{3}}$ | $\boldsymbol{waste}_{\boldsymbol{3}}$ |
| --- | --- | --- | --- | --- | --- | --- | --- |
| 1 | *ngcut8* | 65 | 63 | 3.17% | 86 | 84 | 2.38% |
|  | *ngcut12* | 135 | 127 | 6.30% | 168 | 158 | 6.33% |
|  | *pt8_1_12* | 345 | 328 | 5.18% | 367 | 345 | 6.38% |
|  | *pt8_1_90* | **152** | 148 | 2.70% | **157** | 154 | 1.95% |
|  | *pt8_22_3* | 164 | 160 | 2.50% | 177 | 171 | 3.51% |
|  | average $waste$ | - | - | 3.97% | - | - | 4.11% |
| 2 | *ngcut1* | 69 | 56 | 23.21% | 106 | 86 | 23.26% |
|  | *ngcut2* | 94 | 86 | 9.30% | 178 | 135 | 31.85% |
|  | *ngcut9* | 97 | 92 | 5.43% | 127 | 121 | 4.96% |
|  | *pt6_24_34* | 393 | 375 | 4.80% | 427 | 387 | 10.34% |
|  | *pt8_1_51* | 281 | 264 | 6.44% | 289 | 277 | 4.33% |
|  | average $waste$ | - | - | 9.84% | - | - | 14.95% |
| 3 | *cgcut1* | 86 | 69 | 24.64% | 124 | 109 | 13.76% |
|  | *fran31* | 63 | 52 | 21.15% | 64 | 59 | 8.47% |
|  | *fran32* | 221 | 206 | 7.28% | 252 | 235 | 7.23% |
|  | *fran33* | 167 | 158 | 5.70% | 182 | 165 | 10.30% |
|  | *fran34* | 55 | 51 | 7.84% | 64 | 59 | 8.47% |
|  | average $waste$ | - | - | 13.32% | - | - | 9.65% |
| 4 | *fran41* | 66 | 50 | 32.00% | 68 | 57 | 19.30% |
|  | *pt1_23_52* | **46** | 37 | 24.32% | **49** | 41 | 19.51% |
|  | *pt1_23_94* | 73 | 68 | 7.35% | 80 | 75 | 6.67% |
|  | *pt1_24_15* | 52 | 46 | 13.04% | 55 | 49 | 12.24% |
|  | *pt5_23_32* | 75 | 61 | 22.95% | 77 | 65 | 18.46% |
|  | average $waste$ | - | - | 19.93% | - | - | 15.24% |
| 5 | *C12* | 50 | 47 | 6.38% | 70 | 66 | 6.06% |
|  | *C13* | 47 | 46 | 2.17% | 67 | 64 | 4.69% |
|  | *fran51* | 247 | 238 | 3.78% | 260 | 248 | 4.84% |
|  | *ngcut4* | **52** | 49 | 6.12% | **102** | 75 | 36.00% |
|  | *ngcut7* | **27** | 25 | 8.00% | **36** | 35 | 2.86% |
|  | average $waste$ | - | - | 5.29% | - | - | 10.89% |
| 6 | *C22* | 37 | 34 | 8.82% | 49 | 46 | 6.52% |
|  | *C31* | 56 | 51 | 9.80% | 69 | 64 | 7.81% |
|  | *fran61* | 220 | 208 | 5.77% | 228 | 217 | 5.07% |
|  | *fran62* | 373 | 344 | 8.43% | 385 | 361 | 6.65% |
|  | *ngcut6* | 91 | 84 | 8.33% | 158 | 130 | 21.54% |
|  | average $waste$ | - | - | 9.98% | - | - | 9.52% |
| 7 | *fran71* | 171 | 163 | 4.91% | 182 | 170 | 7.06% |
|  | *fran72* | 201 | 191 | 5.24% | 231 | 219 | 5.48% |
|  | *fran73* | 68 | 58 | 17.24% | 81 | 69 | 17.39% |
|  | *fran74* | 165 | 152 | 8.55% | 183 | 174 | 5.17% |
|  | *fran75* | 453 | 427 | 6.09% | 502 | 447 | 12.30% |
|  | average $waste$ | - | - | 8.41% | - | - | 9.48% |
| 8 | *fran81* | 175 | 163 | 7.36% | 183 | 170 | 7.65% |
|  | *fran82* | 494 | 467 | 5.78% | 546 | 494 | 10.53% |
|  | *fran83* | 262 | 241 | 8.71% | 285 | 272 | 4.78% |
|  | *fran84* | **25** | 23 | 8.70% | **30** | 27 | 11.11% |
|  | *fran85* | 167 | 127 | 31.50% | 206 | 191 | 7.85% |
|  | average $waste$ | - | - | 12.41% | - | - | 8.38% |
| 9 | *C11* | 49 | 47 | 4.26% | 69 | 65 | 6.15% |
|  | *fran91* | 102 | 96 | 6.25% | 115 | 108 | 6.48% |
|  | *fran92* | **83** | 78 | 6.41% | **107** | 99 | 8.08% |
|  | *fran93* | 78 | 64 | 21.88% | 96 | 79 | 21.52% |
|  | *path1* | 137 | 129 | 6.20% | 155 | 144 | 7.64% |
|  | average $waste$ | - | - | 9.00% | - | - | 9.97% |
| 10 | *bwmv155* | 76 | 73 | 4.11% | 87 | 83 | 4.82% |
|  | *bwmv160* | 97 | 93 | 4.30% | 108 | 104 | 3.85% |
|  | *C21* | 37 | 35 | 5.71% | 52 | 48 | 8.33% |
|  | *C32* | 58 | 51 | 13.73% | 70 | 63 | 11.11% |
|  | *gcut13* | 5163 | 4806 | 7.43% | 5201 | 4823 | 7.84% |
|  | average $waste$ | - | - | 7.06% | - | - | 6.73% |
| 11 | *fran111* | 116 | 99 | 17.17% | 182 | 154 | 18.18% |
|  | *fran112* | 109 | 98 | 11.22% | 179 | 151 | 18.54% |
|  | *gcut1* | **734** | 682 | 7.62% | **740** | 696 | 6.32% |
|  | *gcut5* | **1170** | 1116 | 4.84% | **1176** | 1129 | 4.16% |
|  | *gcut9* | 2139 | 2046 | 4.55% | 2145 | 2058 | 4.23% |
|  | average $waste$ | - | - | 9.15% | - | - | 10.29% |
| 12 | *fran121* | 1088 | 1028 | 5.84% | 1123 | 1050 | 6.95% |
|  | *gcut2* | 1242 | 1147 | 8.28% | 1290 | 1173 | 9.97% |
|  | *gcut6* | 2677 | 2516 | 6.40% | 2697 | 2543 | 6.06% |
|  | *gcut10* | 5817 | 5410 | 7.52% | 5997 | 5437 | 10.30% |
|  | *gcut11* | 7149 | 6608 | 8.19% | 7338 | 6644 | 10.45% |
|  | average $waste$ | - | - | 8.48% | - | - | 8.80% |
